# Untangling Computer-Aided Diagnostic System for Screening Diabetic Retinopathy Based on Deep Learning Techniques

**DOI:** 10.3390/s22051803

**Published:** 2022-02-24

**Authors:** Muhammad Shoaib Farooq, Ansif Arooj, Roobaea Alroobaea, Abdullah M. Baqasah, Mohamed Yaseen Jabarulla, Dilbag Singh, Ruhama Sardar

**Affiliations:** 1 Department of Computer Science, School of Systems and Technology, University of Management and Technology, Lahore 54000, Pakistan; f2019288006@umt.edu.pk; 2 Division of Science and Technology, University of Education, Lahore 54000, Pakistan; ansif.arooj@ue.edu.pk; 3 Department of Computer Science, College of Computers and Information Technology, Taif University, P.O. Box 11099, Taif 21944, Saudi Arabia; r.robai@tu.edu.sa; 4 Department of Information Technology, College of Computers and Information Technology, Taif University, P.O. Box 11099, Taif 21944, Saudi Arabia; a.baqasah@tu.edu.sa; 5 School of Electrical Engineering and Computer Science, Gwangju Institute of Science and Technology, Gwangju 61005, Korea; yaseen@gm.gist.ac.kr (M.Y.J.); Dilbagsingh@gist.ac.kr (D.S.)

**Keywords:** diabetic retinopathy, deep learning, deep neural network, automated detection

## Abstract

Diabetic Retinopathy (DR) is a predominant cause of visual impairment and loss. Approximately 285 million worldwide population is affected with diabetes, and one-third of these patients have symptoms of DR. Specifically, it tends to affect the patients with 20 years or more with diabetes, but it can be reduced by early detection and proper treatment. Diagnosis of DR by using manual methods is a time-consuming and expensive task which involves trained ophthalmologists to observe and evaluate DR using digital fundus images of the retina. This study aims to systematically find and analyze high-quality research work for the diagnosis of DR using deep learning approaches. This research comprehends the DR grading, staging protocols and also presents the DR taxonomy. Furthermore, identifies, compares, and investigates the deep learning-based algorithms, techniques, and, methods for classifying DR stages. Various publicly available dataset used for deep learning have also been analyzed and dispensed for descriptive and empirical understanding for real-time DR applications. Our in-depth study shows that in the last few years there has been an increasing inclination towards deep learning approaches. 35% of the studies have used Convolutional Neural Networks (CNNs), 26% implemented the Ensemble CNN (ECNN) and, 13% Deep Neural Networks (DNN) are amongst the most used algorithms for the DR classification. Thus using the deep learning algorithms for DR diagnostics have future research potential for DR early detection and prevention based solution.

## 1. Introduction

**Figure 1:**
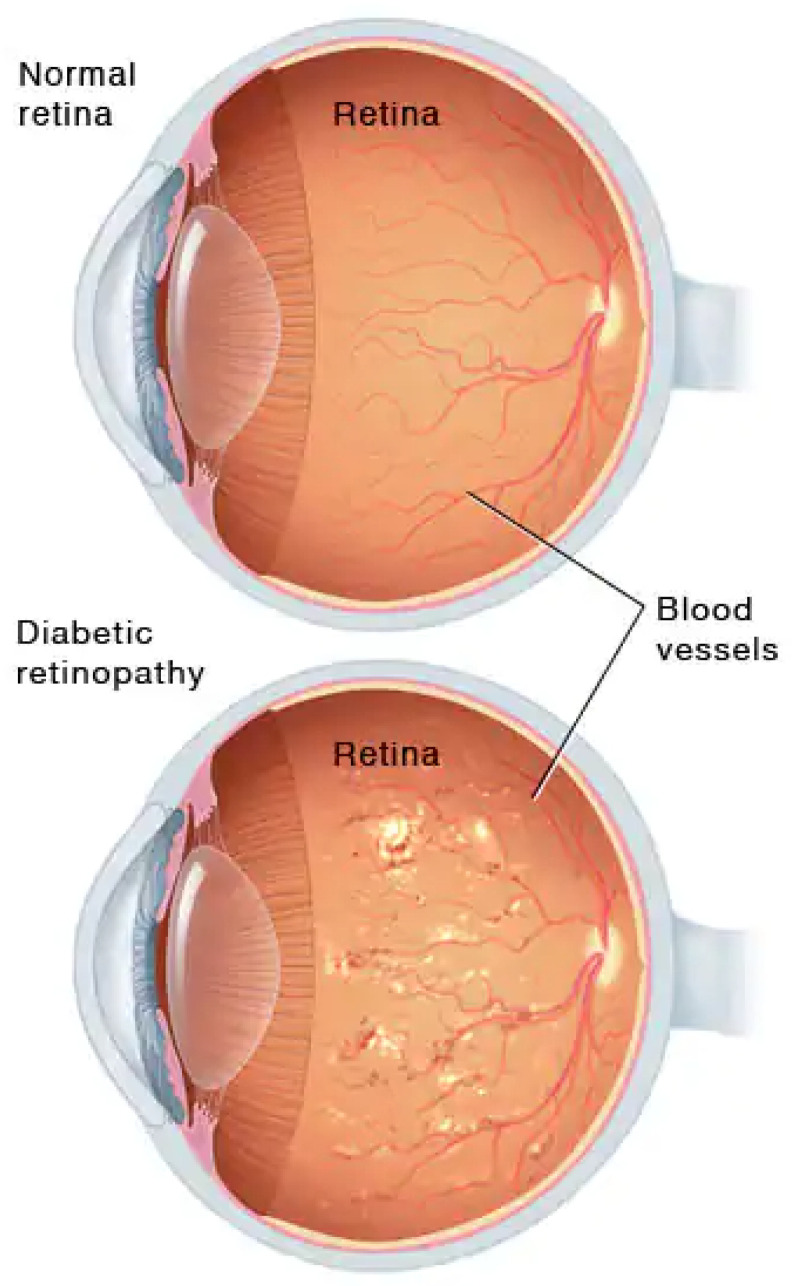
Comparison between Normal and DR eye [2].

Diabetes, also called diabetes mellitus, is a disease that occurs when the human body produces a large amount of blood glucose. Glucose a the main source of energy and comes from the food that the human body consumes daily. Diabetes has become the cause of many diseases such as heart, stroke, nerve damage, foot problems, gum disease, and many more [1]. The eye is also one of the major organs affected by diabetes. The diabetic problem associated with the eye is called Diabetic Retinopathy (DR). Comparison of a normal and DR eye is illustrated in Figure 1 inspired by [2]. DR is the primary cause of blindness, mostly in adults [3]. It disturbs the retina, a light-sensitive part of the eye, and can become the cause of blindness if it has not been diagnosed in the early stages, or not treated well. Chances of suffering DR become higher if the duration of having the disease is extensively over the threshold. A patient with a history of 20 years of diabetes has an 80% chance to encounter DR [4]. It is also eminent that the patient of DR may have no symptoms or only have a mild vision problem. Any damage or abnormal changes that occurred in the tissue of the organ caused by the disease is called a lesion. A consensus of international experts [5] formulated International Clinical Diabetic Retinopathy (ICDR) and Diabetic Macular Edema (DME) Disease Severity Scales to simply standardize the DR classification. ICDR improves the coordination and communication among physicians for caring for patients with diabetes [6,7].

### 1.1. Retinal Lesions

Microaneurysms (MA), Hemorrhages (HM), and soft exudates and hard exudates (SE and HE) were frequently observed by ophthalmologists in the retinal fundus images [8]. The presence of these lesions are pathognomonic signs of Diabetic Retinopathy (DR).

**Figure 2:**
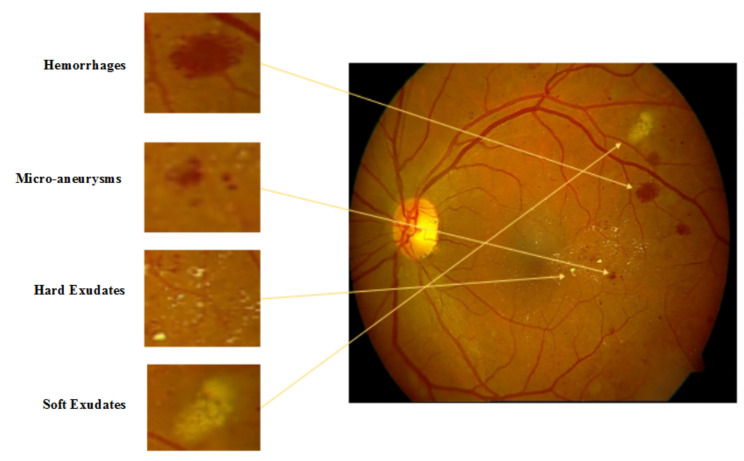
Lesions in retinal fundus image.

(i) Microaneurysms: The earliest symptoms of DR is the presence of microaneurysms in the retina of diabetic patient. They may present in the group of tiny, dark red spots or look like tiny hemorrhages within a retinal light-sensitive area. They vary in size, mostly between 10 to 100 microns but less than 150 microns [8,9,10]. In the early stage, it is not threatening to the eyesight but needs to be diagnosed and treated early.

(ii) Hemorrhages: The primary symptom of DR is leakage of blood in the retina of the eye that may appear anywhere in the retina, with any shape and size [11]. Hemorrhages are of many types such as flame hemorrhages or dot and blot hemorrhages.

(iii) Soft Exudates: Soft exudates are also known as cotton wool spots [12]. These are often pale yellow or buttery in color and round or oval-shaped due to capillary occlusions that caused permanent damage to the function of the retina.

(iv) Hard Exudates: This is the most advanced abnormality of the DR. These can be in different sizes from small tiny fragments to larger reinforcements and become the cause of visual impairment by foiling light from reaching the retina [8]. Figure 2 demonstrates the lesions in retinal images.

Automatic detection of these lesions could help in early DR monitoring and control of progression in an efficient and optimal way. For many decades researchers have put their efforts towards building an effective method through which they can identify different abnormalities by achieving higher accuracy. However, extracting accurate features is a challenging task. Moreover, the selection of accurate machine learning (ML) algorithms at early stages lead to better results in DR early detection and thus can consider the foremost step of pervasive solutions. All algorithms have their pros and cons, but no method can be regarded as superior in all stages [13]. Recently, deep learning networks have achieved great attention due to their ability to automatically extract features and strong abstract representation learning. Like machine learning, this paradigm is also based on learning from the data, but instead of using handcrafted features, which is a very difficult and time-consuming task, DL provides many advantages such as improvement in the performance of training data and the exclusion of the clumsy feature extraction process.

With the huge development of computing power and digital imaging, the algorithmic revolution to detect the severity of DR has improved over the past 5 years. A significant amount of work has been already achieved for automatic DR detection with the use of fundus photographs, but only one study has been procured to date [14]. Previous studies and reviews have discussed traditional methods of DR detection [9,15,16], but expert knowledge is required for hand-engineered characteristics of this highly sensitive investigation with several parameter settings. The last era of computer-based medical applications and solutions has provided a huge amount of data and the hardware capabilities to process these dataset. For instance, intensive increases in graphics processing units (GPUs) have encouraged many researchers to apply computer vision and image processing to achieve optimal performance. These operations have gained the interest of many researchers and market contributors over traditional methods. The major categorization of the work completed in this paper is divided into three main sections: *(i)* overview of different tasks to diagnose DR, *(ii)* preparation of a summary of publicly available dataset, and *(iii)* providing an outline of DL methods for blood vessels, Optic disc segmentation, detection, and classification of various DR lesions. However, this review does not embody the queries we tend to address during this study. A comparison of these studies has been presented in Table 1.

### 1.2. Purpose of This Study

This article presents a descriptive review to explore the significant work related to detecting DR by classifying different stages of DR, for instance mild, severe, and PDR, by using deep learning techniques. The proposed study also focuses on the public as well as private dataset used in the primary selected studies. Although in the past several literature reviews have been presented for compiling the deep learning in DR detection [14,19,20], some limitations have been found in these studies; these are being addressed in this study.

*Contributions:* This study is piloted systematically and follows the practice of the Cochrane methodology to synthesize the evidence by randomized trials for addressing the research questions [21]. The primary aim of this study is to provide the following state-of-the-art features:

- A comprehensive overview of DR grading protocols and taxonomy to provide a general understanding of DR.
- To identify the need for automatic detection of DR and ascertain how deep learning can ease the traditional detection process for early detection and faster recovery.
- To conduct the meta-analysis of 61 research studies addressing the Deep learning techniques for DR classification problems and quality assessment evaluation of these studies.
- To identify the quality research gaps in the body of knowledge in order to recommend the research directions to address these identified gaps. To the best of our knowledge, this study is the first work that focuses on preparing the taxonomy of DR problems and identification of the deep learning techniques for DR classifications based on the grading protocols that have efficient and highly accurate results.

The rest of the paper has been structured in the following manner: Section 2 outlines the methodology used to conduct this study. It includes three phases: planning, conducting, and documenting; moreover, the classification criteria to categorize studies according to scoring criteria is also part of this section. Section 3 describes the mapping study and findings which discussed the most used deep learning algorithms as well as discussing publicly available dataset to diagnose DR stages. Section 4 includes the discussion section and explains the validity threats that could affect this study. The last section, Section 6, provides a conclusion with suggestions and future work.

## 2. Research Methodologies

### 2.1. Systematic Literature Review Protocol

**Figure 3:**
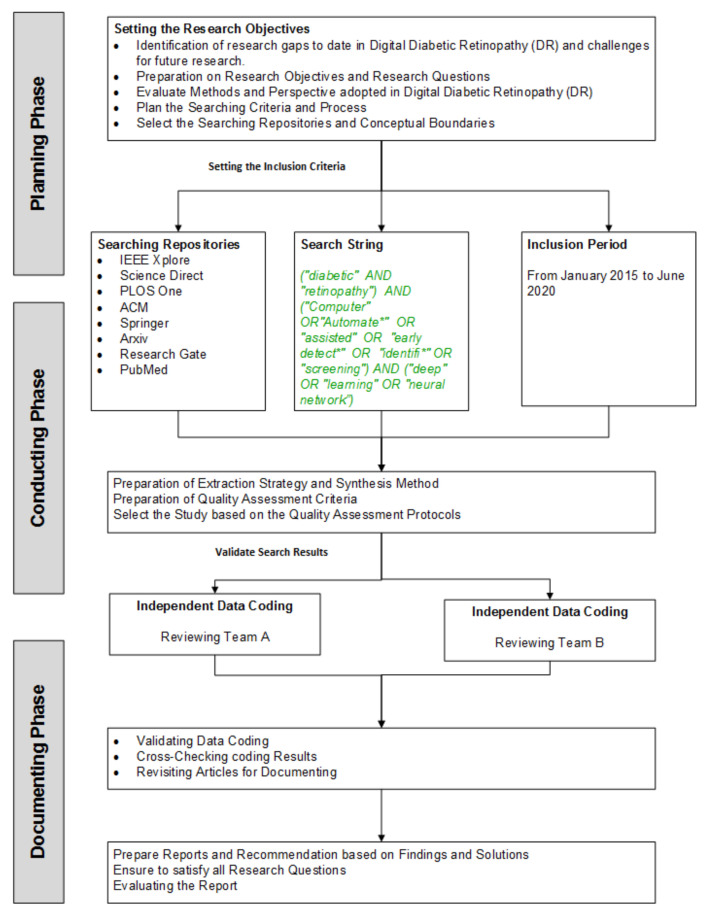
Systematic Literature Review Methodology.

We have developed a research protocol for identifying, planning, synthesizing, and screening all the published work in the proposed research questions of this review. The main idea of this review is to discover the body of knowledge for using deep learning in DR. For this review, we have adopted the guidelines of the Cochrane methodology as a reference [21]. According to the author, the study is composed of three basic steps (i) planning, (ii) conducting, and (iii) documenting the review. Thus, we have developed the research protocol inspired by the their methodology, presented in Figure 3. The main objective of our study is designated to explore well-reputed digital libraries for perusing the answers to research questions using search terms.

### 2.2. Planning Phase

The initial phase of the study is to devise the protocol for review in a rigorous and well-described manner to preserve the primary rationale of the study. In this phase, we have prepared the review procedure from identification, evaluation, and interpretation of all available resources for deep learning in DR. Moreover, research objectives, research questions, and searching processes have been the main segments of this part of the research.

#### 2.2.1. Research Objectives

The objectives of building this study are:

- RO1: To identify the clinical features of retinal images for DR detection.
- RO2: To consolidate the DR stages and grading protocols for DR classification.
- RO3: To propose a state-of-the-art taxonomy to highlight the deep learning methods, approaches, and techniques used in DR.
- Investigation of deep learning methods used to detect DR.
- RO4: To consolidate the available dataset of DR at well-reputed repositories for validating the deep learning approaches to classify different DR stages.
- RO5: To identify the research gaps in terms of challenges and open issues and propose the solution in each DR research domain.

#### 2.2.2. Research Questions

In order to gain an in-depth view of the domain, the Research Questions (RQ) have been presented in a Table 2 with their resultant motivations. These questions will help us in classifying the current research in detecting DR by deep learning and also provide the future directions of the research domain.

#### 2.2.3. Search Process and String

**Figure 4:**
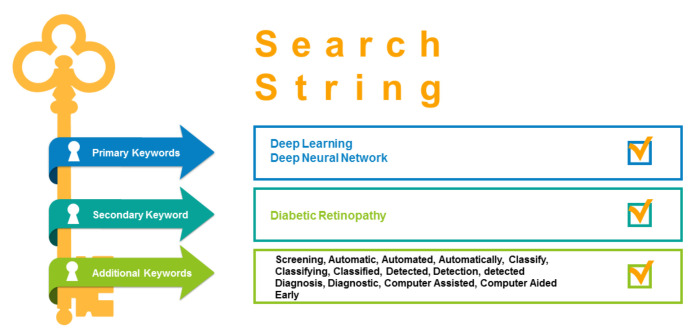
Target combination of words to form Search String.

There is a need for a well-managed plan to prepare for the searching process and narrow down the targeted results for focused solutions and answers. For the effective resultant approach, we have executed automatic and manual searching strategies from well-reputed repositories such as IEEE Xplore, Science Direct, Arxiv, ResearchGate, ACM, LANCET, PLOS, PubMed, and SpringerLink. After a closed and rigorous review, the following search string has been prepared for automatic retrieval of search studies from the selected search engines and demonstrated in Figure 4 as:

("diabetic" AND "retinopathy") AND ("Computer" OR "Automate*" OR "assisted" OR "early detect*" OR "identifi*" OR "screening") AND ("deep" OR "learning" OR "neural network”)

However, to retrieve the most relevant research papers, we have performed the primary research retrieval on selected search engines to find adequate results. Furthermore, for optimal retrieval, the search terms need to be bound, because many research terms can falsify the results due to the extended term of machine learning as a base. Therefore, there is a need to limit the coverage of the term to deep learning only. The search conditions for the searching process have been described as:

- Retrieval of relevant keywords in deep learning for DR that can satisfy all research questions of this study;
- Identification of domain synonyms and related terms;
- Formulation of state of the art search string consisting of a key and substantial terms with “AND” or “OR” Boolean operators.

### 2.3. Conducting Phase

The second phase of the research methodology of this study is the execution of the search. It deals with the collection of all the existing research papers and studies in deep learning for DR. However, before the systematic literature review, we have created the inclusion and exclusion criteria for the study with data extraction and synthesis strategy.

#### 2.3.1. Inclusion/ Exclusion Criteria

The preliminary step after selecting all papers from well-reputed repositories and search engines is to eliminate redundant titles and all irrelevant papers. Based on Table 3, the retrieved papers that follow any of the exclusion criteria have been excluded.

#### 2.3.2. Study Selection

This study aimed to identify all potential papers that were the most significant to the objectives of this systematic review. To eliminate redundancy of inclusion, we have ensured to consider the papers once rather than they be retrieved from different search engines or repositories. Every selected paper was reviewed based on reviewing the title, abstract, keywords, and conclusion. Table 4 demonstrates the details about the number of searchers found in the digital libraries and selected research papers after applying inclusion/exclusion criteria.

#### 2.3.3. Quality Assessment Criteria

In the final set, each publication was assessed for its quality. The quality assessment was performed during the data extraction phase and ensures that selected studies must be a valuable contribution to the study. Hence, to fulfill this purpose, a questionnaire inspired by [22] was designed. These criteria are labeled as (a), (b), (c), (d), and (e). The final score of each paper is calculated by assigning the individual score for these criteria and accumulative in last. The quality assessment and scoring criteria are represented in Table 5.

#### 2.3.4. Data Extraction Strategy and Syntheses Method

Data extraction strategy has been defined in Table A1 in Appendix A to collect the relevant information which was required to address research questions defined in this systematic mapping study. Data extraction ID from DE1 to DE3 collects the basic information related to the papers. These data extraction features include study identifier, publication type, source of the publications, and the publication location. The remaining IDs from DE4 to DE6 were extracted after studying the papers.

**Figure 5:**
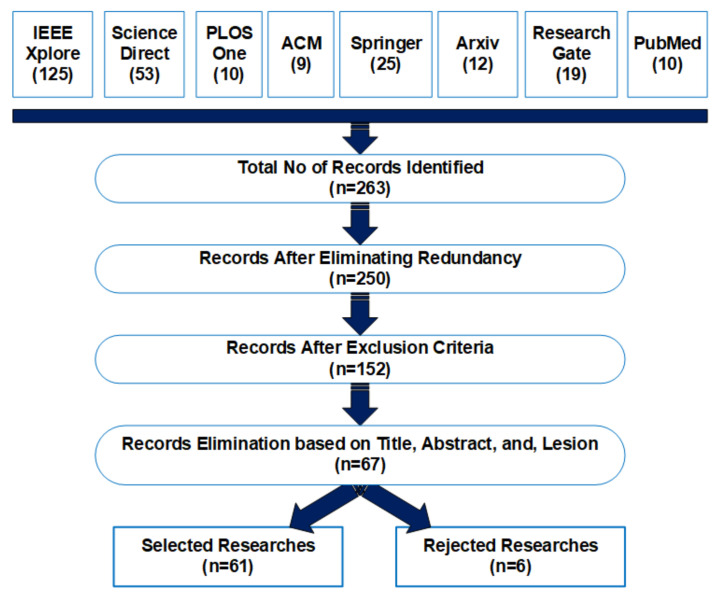
Studies Selection Process and Results.

**Figure 6:**
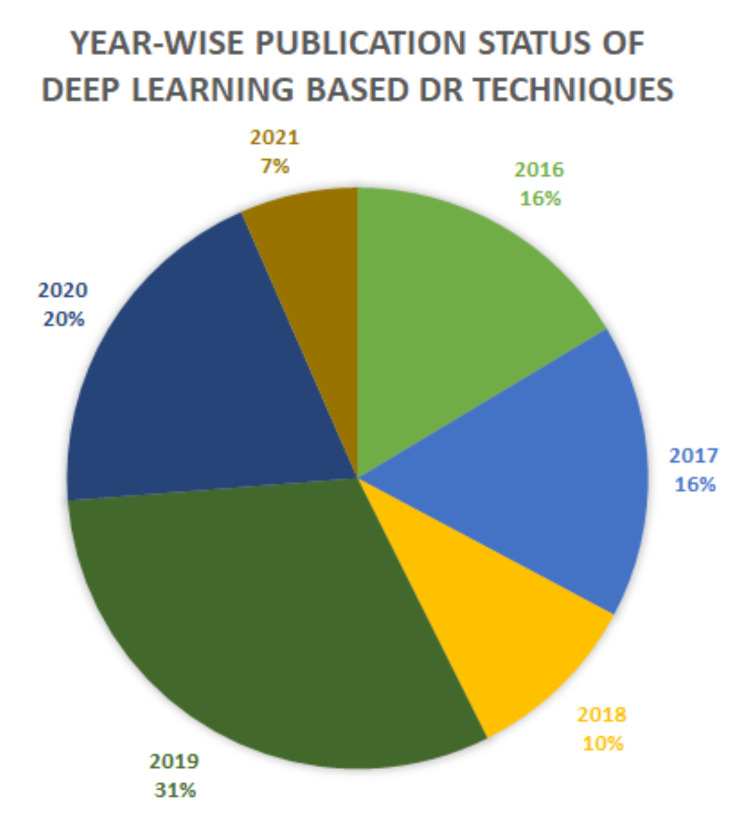
Year-wise Publication Results.

Figure 5 shows the step by step procedure for searching the relevant studies for this study. We retrieved a total of 5120 studies after searching in the selected digital libraries. In the first step, we eliminated studies that were not relevant to the research questions as listed earlier and have also removed redundancy. After the initial step, rigorous manual screening based on the title, abstract, and content of initial screened studies was performed. The studies focused only on detecting lesions of diabetic retinopathy resulted in the exclusion of 755 studies. In the last step, only 61 studies were selected for further consideration. Importantly, 90% of the selected studies were published in JCR-listed journals and 10% in proceeding conferences. Figure 6 represents the year-wise publication status with 16% papers selected from 2016 and 2017, 10% from 2018, 31% from 2019, and 20% from 2021.

#### 2.3.5. Classification Criteria

The classification criteria were divided into categories that were established through selected primary studies as below.

RQ1 categories include:

- Deep learning techniques to identify DR severity levels or stages;
- Stages of DR;
- Highest achieved an accuracy rate of deep learning algorithms.

RQ2 categories include:

- Publicly available dataset;
- Privately available dataset.

And the last question RQ3 includes

- Limitation of the study;
- Open issues and challenges;
- Future Directions.

### 2.4. Documenting Phase

After conducting the study of the study, we presented the analysis of the results in a comprehensive format and also developed the meta-analysis in Table 6, Table 7, Table 8 and Table 9 in order to present the information in a precise, comprehensive, and easy to understand format.

## 3. Mapping Results and Findings

**Figure A1:**
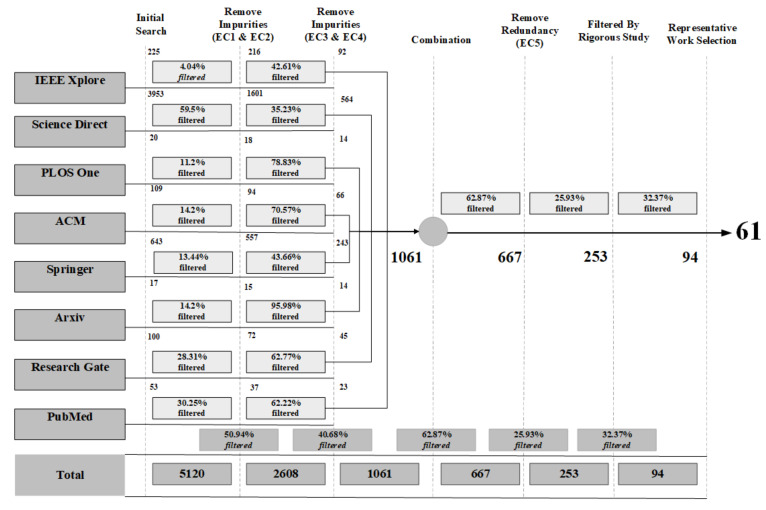
The obtained results from the execution of the study filtering process.

The step wise process of examination, review, and extraction is presented in Figure 5 and Figure A1. These figures demonstrated step by step observation and assessment of selected studies. After profound examination, six more papers were eliminated due to less relevancy with the proposed research questions of this study. Finally, 61 studies were selected after automatic and manual screening and evaluation. This section aims to review, analyze, and present the selected studies to answer the research questions listed in Table 2. Each research question is addressed individually along with the in-depth review and study. Table 10 and Table 11 represent the quality assessment of each selected paper with respect to the quality assessment criteria presented in Table 5. The results show the area of study and research has potential for future enhancements and optimization in health care solutions concerning image processing and deep learning.

### 3.1. Research Question 1: What Clinical Features of Retinal Images Are Required for DR Detection and Classification and Which Deep Learning Methods Are Mostly Used to Classify DR Problems?

**Figure 7:**
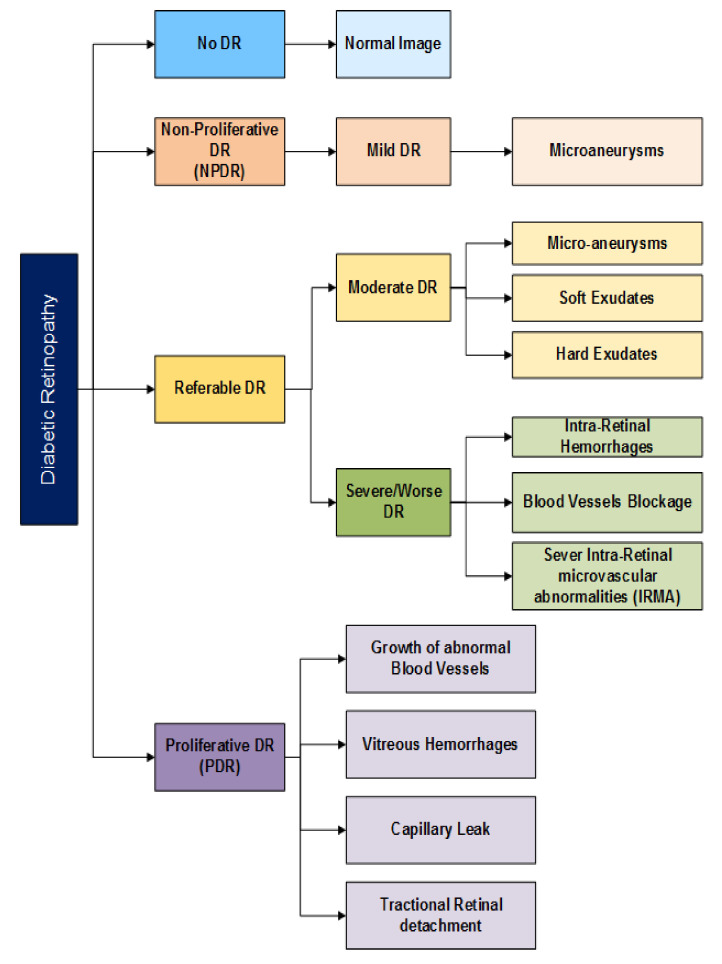
Taxonomy of Diabetic Retinopathy (DR).

To answer RQ1, deep learning techniques have been reviewed that were used to detect DR in its early stages. Furthermore, architecture, tools, libraries, and performance matrices have been analyzed and reviewed. However, before adding the description of all deep learning methods, there is a need to understand the clinical features and taxonomy required to detect DR. In the subsection below, we have comprehended some of the important DR clinical features and have also presented the Lesions and DR grading protocols. Furthermore, the taxonomy of DR detection classification is demonstrated in Figure 7.

#### 3.1.1. DR Stages and Grading

**Figure 8:**
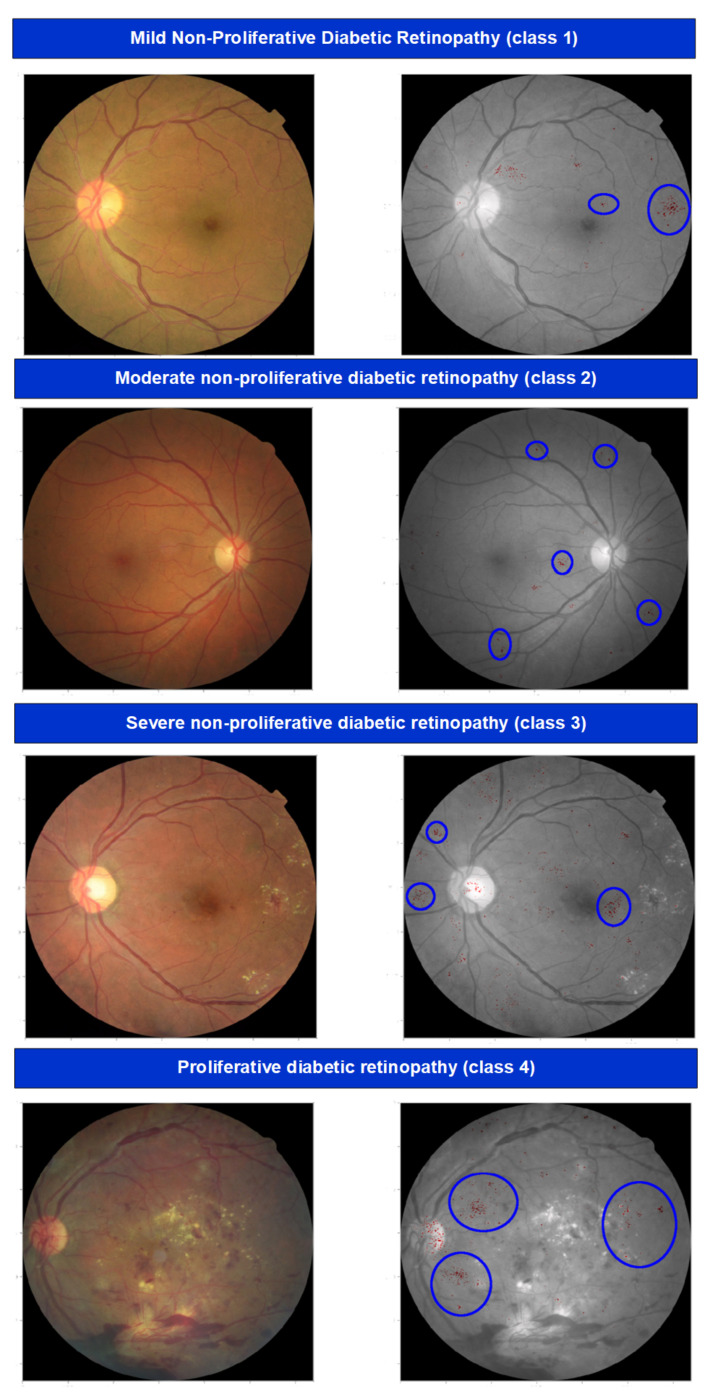
Five stages of Diabetic Retinopathy.

**Figure 9:**
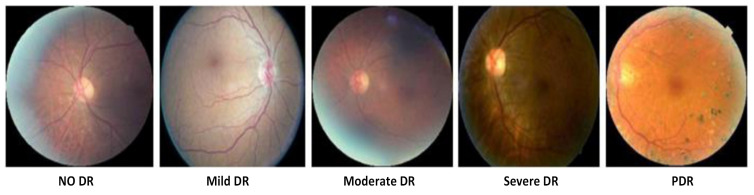
Five stages of Diabetic Retinopathy.

According to the lesions, the severity of DR is mainly divided into *five* stages [17] and illustrated in Figure 8 and Figure 9:

- No DR;
- Moderate non-proliferative DR (Class 1);
- Mild non-proliferative DR (Class 2);
- proliferative DR (Class 3);
- Severe DR (Class 4).

In no DR, an eye is clear from any problem caused by DR. In mild diabetic retinopathy, the retina of the eye has a balloon-like swelling called micro-aneurysms, and this is the early stage of the disease. As the disease progresses, it converts into a more severe stage called moderate diabetic retinopathy in which blood vessels nurturing the retina of the eye may swell and distort, thus causing the retina to lose its ability to transport blood and become the reason for the change in the appearance of the retina. In severe DR, many blood vessels have been blocked, causing non-supply of blood to the retinal areas.

The most advanced stage of the DR is Proliferative Diabetic Retinopathy (PDR) and it may cause sudden blindness due to the vitreous hemorrhages of the central hemorrhages. If DR is not detected and treated properly in its Severe DR stages, it could turn into PDR [13]. Demonstrations of different stages of DR are shown in Figure 7 and Figure 8 presents the taxonomy of DR concerning the severity of the problem. The summary of diabetic retinopathy grading protocols is illustrated in Table 12 and nomenclature is presented in Abbreviations.

#### 3.1.2. DR Screening & Detection Techniques

Detection and diagnosis of DR in its early stage can help to prevent and slow down the process of visual impairment or blindness. Expert ophthalmologists use standard grading protocols for grading diabetic retinopathy. The screening method can help to enable DR detection earlier. In the screening session, retinas of both eyes are observed by expert ophthalmologists, and if the DR is detected, the patient is referred for further treatment [83]. The DR screening methods are enlisted hereunder:

- Visual Activity Test;
- Ophthalmoscopy or fundus photography;
- Fundus Fluorescein angiography (FFA);
- Retinal vessel analysis;
- Optical coherence tomography (OCT).

#### 3.1.3. Clinical Features of Retinal image for DR Detection

(1) Retinal Blood Vessel Classification

Retina blood vessels are the anatomical and complex structure of the human eye and only be observable by the non-invasive method. The structure of these vessels and their changes reflect the impact of DR stages and help to understand the prodigality of the disease. Moreover, a cataract is an overcast and thick area of the eye. It starts with the protein transformation in the eye which makes the image of any object foggy or frosty, and also causes pain. It is graded into four classes, normal, mild, moderate, and severe [82]. Based on the cataract clinical understanding, the vessel segmentation and classification is tedious work due to several features of vessels such as the length, width, position, and tortuosity of branches. Therefore, automatic DR detection for vessel segment understanding and classification is necessary for DR early detection and recovery. Several deep learning studies have been undertaken to achieve accurate and better methods and models concerning retinal blood vessel classification. For instance, Deep Supervision and Smoothness Regularization Network (DSSRN) was proposed for deep observation of the regular network of blood vessels. DSSRN was established by VGG16 and Conditional Random Fields were used to check the smoothness of the results with higher accuracy but minimum sensitivity [84]. Patch-based ensemble models have been used on DRIVE, STARE, and CHASE models with optimal accuracy and sensitivity. Moreover, there are several classification methods used for understanding the transference of semantic knowledge between the output layers of sides. The ResNet-101 and VGGNet CNN models were used for experiments and have achieved better accuracy on STARE and Drive Dataset. However, these models cannot be used for micro-vessel segmentation with wider angles [50,64]. Moreover, in another study, the CNN model was integrated with 12 variations to differentiate vessel and non-vessel segmentation. DRIVE dataset was used for process evaluation [84]. DCNN was used on three dataset, DRIVE, STARE, and CHASE, for vessel segmentation by extensive pre-processing features, i.e., normalization and data augmentation by geometric feature transformation [65]. Regression Analysis was applied with VGG pre-trained model on vessel images by modifying VGG layers on DRIVE and STARE dataset [85]. The performance analysis of all the proposed segmentation models is presented in Table 6, Table 7, Table 8 and Table 9.

(2) Optic Disc Feature Segmentation

The optic disc feature can be extracted by considering the conversion and contrast as parameters and then developing the DR diagnostic algorithm. Importantly, localization and classification of optic disc segmentation are two well-known operations to detect DR. During the survey of this study, it has been observed that boundary classification, edge detection, and orbital approximation are major tasks in DR deep learning optic disc feature classification. It has been observed that patch-based CNN, multi-scale CNN, FCN, and RCNN are majorly used deep learning methods for segmentation and localization. The detailed description and performance analysis of this feature is presented in Table 6, Table 7, Table 8 and Table 9.

(3) Lesion Detection and Classification

Referable DR identification and classification is not possible without information about lesions. However, state-of-the-art machines can identify DR without lesion information, and thus their predictions dearth clinical clarification rather than having high accuracy. However, it has been observed that recent enhancements in visualization methods have encouraged researchers to work intensely in DR detection. Thus, generic heat maps and gradient models are a major contribution to lesion detection and classification to date. Hence, multiple lesions have still not achieved acceptable accuracy. During the study, we intensively reviewed lesion-based DR. In general, we retrieved 23 papers for DR lesion classification explaining the micro-aneurysms (11 studies), hard exudates (9 articles), and macular edema and hemorrhages (3 studies). The details of these studies are described in Table 6, Table 7, Table 8 and Table 9.

#### 3.1.4. Deep Learning Methods for DR Problems

(1) Convolutional Neural Network (CNN)

Convolutional neural network (CNN) is a category of neural networks heavily used in the field of computer vision. It consists of hidden, input, and fully connected output layers. It derives its name from the convolutional layer, which is part of the hidden layer. The hidden layer of the CNN typically consists of convolutional, pooling, normalization, and fully connected layers [54,86,87]. The convolutional layer is the main building blocks of any CNN architecture. CNN is the most common type of architecture used in detecting DR in its early stages due to its neural and hierarchical structure. Authors in [23,29,39,43,88] demonstrate the effectiveness of CNN for five-stage classification of DR, and authors in [39,40,44] demonstrate CNN’s performance parameters for classifying two or three stages of DR. Refs. [46,51,54,57,60] have used the CNN architecture either to improve the results of already published studies or to detect different five stages of DR [51,53,54,58,59,69,77,89]. Experiments with a small fraction of data can achieve state-of-the-art performance in referable or non-referable DR classification.

(2) Deep Neural Network (DNN)

DNN has also shown a remarkable performance in many computer vision tasks. The term “deep” generally refers to the use of a few or several convolutional layers in a network, and deep learning refers to the use of the methodologies to train the model to learn essential parameters automatically using data representation to solve a specific domain of area of interest [10,55,72,90]. The major benefit of this algorithm is that with the increased number of samples in the training set, classification accuracy also increases. This algorithm has been used in [25,30,35,44,46,49,51] to improve classification accuracy among different DR stages. In [40], the author builds a DCNN model with three fully connected layers to automatically learn the image’s local features and to generate a classification model.

(3) Ensemble Convolutional Neural Network

An ensemble of two or more different techniques to find better training results is not a new technique in machine learning. For example, upgrading the decision tree model to the random forest model. In deep learning, an ensemble of neural networks is performed by collecting different networks with the same configuration, and initial weights are assigned randomly to train some dataset. Every model makes its predictions. In the end, the actual prediction is calculated by taking the average of all predictions. The number of models in the ensemble method is kept as small as three, five, or ten trained models due to the computational cost and due to the lessening returns in performance. Moreover, ensemble techniques have been used to improve classification accuracy and model performance [14,26,32,37,38,50,53,55,63]. Based on the primary selected studies, some other DL methods have been applied to detect a DR problem by using different data sets. These algorithms are graph neural network, Hopfield neural network, deep multiple instance learning, BNCNN, GoogleNet neural network, and densely connected neural network.

**Figure 10:**
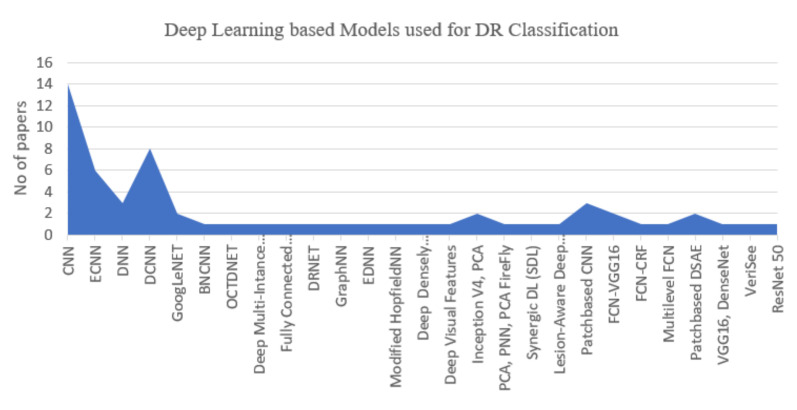
Deep Learning based Models used for DR Classification.

Figure 10 helps us to answer RQ1. The chart shows that researchers mostly prefer to use CNN architecture (31%) for detecting complex stages of DR. On the other hand, Deep CNN (20%) also gains good attention and results as well. Based on the final selected studies, three major algorithms CNN, DCNN, and ensemble CNN, were commonly implemented for DR detection. Moreover, for red-eye lesion detection, YOLOv3 has also been proposed [76] by using CNN and DCNN. Deep belief networks, Recurrent Neural Networks (RNN), autoencoder-based methods, and stacked autoencoder methods have also been used for DR classification problems. However, some researchers [26,27,28,32,37,46,63], ensemble CNN variations to achieve better performance and results. The author in [35] used the Adaboost algorithm to combine ensemble CNN models and presented the proven optimization with higher accuracy. Moreover, the authors in [40,50,53,60,61,73,80,91,92] tend to combine two or more CNN models to empower the training process in the field to detect DR. The in-depth analysis with major contribution of each selected study has been presented in Table 9Table 10.

### 3.2. Research Question 2: Which DR dataset Have Been Acquired, Managed, and Classified to Identify Several Stages of DR?

**Figure 11:**
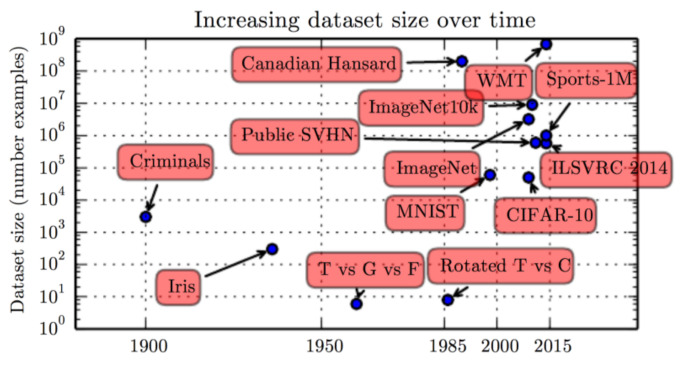
Dataset Size Over Time [87].

To answer RQ2, we thoroughly studied each final selected study. Each of the 61 studies used different sets of private or publicly available data to validate their results. This section started with reviewing the most popular publicly available dataset for the task of detecting different severity levels of DR. Thereafter, we focus especially on those dataset those were used in the selected studies by the researchers. Figure 11 represents the rapid increase in the DR data set for classification and segmentation [87].

#### Publicly Available Dataset

There are many data sets publicly available online, according to the eye conditions such as glaucoma, age-related macular degeneration, and diabetic retinopathy. These publicly available dataset aim to provide good retinal images for training and testing purposes. These dataset are very important for the enhancement of science as they lead to the development of better algorithms as well as support technology to transfer from research laboratories to clinical practice. The descriptions of these dataset are enlisted in Table 13. Moreover, there are some reasons for providing dataset publicly. (1) Sharing and availability of data will encourage researchers to process and analyze said dataset, thus will lead to better solutions in DR-based applications. Diversity and availability of dataset can have remarkable results. In previous studies, it has been observed that, due to the unavailability of relevant data, many solutions were not able to achieve their appropriate goals. However, these solutions can have ultimate results by associating with the relevant and updated dataset. (2) Almost all kinds of research in the same area of study require the same type of data; it would not be necessary to replicate data if these dataset are available publicly and can be beneficial in an economic and financial term, as well as saving time and effort. (3) Publicly available data and their re-processing and re-use for diverse analyses are fundamental resources for innovation. The research and experiments on these dataset can provide new insights and opportunities for researchers to collaborate with medical practitioners to develop potential and data-driven applications. Moreover, these dataset will statistically empower the solutions and encourage multidisciplinary researchers to come up with new analyses by using their expertise, thus leading to comparative analyses and optimal solutions.

(i) DRIVE Dataset: DRIVE abbreviated as Digital Retinal Images for Vessel Extraction (DRIVE) is a publicly available dataset, which consists of 40 color fundus images used for any automated vessel detection algorithms. DRIVE images were taken from the Netherlands during the DR screening program. The total screening population consists of 400 patients aged 25–90 years. From these data, 40 images were selected randomly; 33 images showed no signs of diabetic retinopathy but 7 images showed early signs of DR such as exudates and hemorrhages. Each image is in the form of a JPEG compress. All these images were acquired using a canon CR5 camera. Moreover, the set of these 40 images was equally divided into 20 images for training and the other 20 images for test data. For the training set, a single manual segmentation is available for the test dataset and two manual segmentation are available for vasculature. This process of the extraction of the blood vessel or segmentation from the retinal image is the task many researchers tried to automate as it is a very difficult and time-consuming process. The studies [37,64,65,67,68,69,70,73,73] used the DRIVE dataset for their research work.

(ii) STARE Dataset: Structured Analysis of the Retina (STARE) was initiated by Micheal Goldbaum at the University of California. It contains 20 digitized images captured with TopCon TRV 50. The STARE website provides 20 images for blood vessel detection with labeled ground truth and 81 images for optic disc localization with ground truth. The performance of the vessel detection is measured using ROC curve analysis, and where sensitivity is the correct, classified proportion of blood vessels and specificity is the proportion of correctly classified normal pixels. In the case of the optic disc, localization performance is measured against the correctly localized optic disc, and localization is successful if the algorithm detects optic disc within 60 pixels from the ground truth [27,64,65,68,72] have used the STARE dataset for results validation.

(iii) MESSIDOR Dataset: Messidor is the largest dataset publicly available online that contains 1200 eye fundus color retinal images. It was acquired by the three ophthalmology departments using a color video 3CCD camera. All images are stored in the format of TIFF. Eight hundred images were acquired with the dilation of the pupil and the other 400 were without dilation. Its primary aim is to analyze the complexity of diabetic retinopathy by evaluation and comparison of algorithms. The severity of DR is measured based on the existence and number of diabetic lesions and also from their distance to the macula. Many studies for instance [10,32,35,39,40,45,46,57,58,59,65,68,70,79,89] have used the Messidor dataset for deep learning and classification.

(iv) DIARETDB Dataset: This is a publicly available dataset for the detection of diabetic retinopathy from fundus images. The current dataset consists of 130 images from which 20 images show no sign of retinopathy but the remaining show signs such as exudates, hemorrhages, and micro-aneurysms. All images were captured using a 50-degree field of a view fundus camera with unknown camera settings. The main aims of designing this dataset are to define data unambiguously and testing, which can be used as a benchmark for diabetic retinopathy detection methods. DIARETDB has a further three levels DIARETDB 1, DIARETDB 2, and DIARETDB. Refs. [32,60] used DIARETDR dataset.

(v) EYEPACS Dataset: EyePACS consist of nearly 10,000 retinal images and provides a free platform for retinopathy screening. All images were taken under a different variety of retinal conditions. Every subject provides the left and right fields. The clinician-rated each image according to the presence of diabetic retinopathy. Images for this dataset are taken from different type and model of camera. Due to the large size of the dataset, it has been divided into separate files with multi-part archives such as train.zip, test.zip, sample.zip, etc. [10,40,46,58,65,68,70,78] have used this dataset in research.

(vi) KAGGLE Dataset: The dataset (Kaggle, 2015) by Kaggle consists 82GB image files, which contain a total of 88,702 color fundus images. Each image was rated by an ophthalmologist for the presence of lesions, using severity levels from 0 to 4. Level 0 contains images with no sign of retinopathy and level 4 shows images with advanced stages of retinopathy. The training set is highly unbalanced, with 25,811 images for level 0, 2444 images for level 1, 5293 images for level 2, 874 images for level 3, and 709 images for level 4. This dataset contains challenging images, which are of poor quality and unfocused, which makes it difficult for any algorithm to classify them correctly according to the severity of retinopathy. Many researchers, for instance, [10,22,26,32,35,40,44,46,53,77,90] have used the Kaggle dataset.

(vii) Others: Instead of using only publicly available dataset, some researchers [23,24,28,29,30,31,33,34,36,37,39,40,47,55,60,72,74,80,81,93] have preferred to not use public dataset to classify DR stages. And few researchers [40,50] have used two dataset, one public and another private, to create their new dataset. To answer RQ3, section A provides detailed information about the existing dataset that was mostly used to detect two to five stages of DR. Figure 8 shows that, other than only using public dataset, some researchers prefer to collect their dataset (34%) either by collecting from the screening process or different hospitals or research laboratories. The Kaggle dataset (29%) also gains more attention to detect all five stages of DR. In 20% of the cases Messidor, 9% EyePACS, and only 2% DIARETDR, STARE, and DRIVE, dataset were used. Table 13 shows some of the publicly available dataset description format and dataset download links.

### 3.3. Research Question 3: What Are the Open Issues and Challenges of Using Deep Learning Methods to Detect DR?

RQ3 is associated with finding the main challenges faced by the researchers while conducting experiments further highlighted the open issues of DL in the area of detecting DR automatically.

#### 3.3.1. DR Detection Challenges

After thoroughly reviewing and processing the final selected papers, we have highlighted some of the main challenges faced by the researchers. Generally, these challenges include the size of the dataset, dataset bias, dataset quality, computation cost, and ethical and legal issues.

(i) Dataset Bias: The annotation work is based on the experience of the trained ophthalmologists or physicians on how they grade the images [28,40,58,83]. As a result, the algorithm may perform differently when an image with missing variables (microaneurysms, exudates, as well as important biomarkers of DR) that the majority of clinicians could not identify fed into the network, which may lead to various errors in the results [20,30,42,46,94].

(ii) Dataset Quality Issue: The quality of the DR imaging dataset is mainly affected by how the dataset is collected, i.e., quality of the camera, type of the camera, and overexposure to light [33]. A low-quality camera may be missing important information, and dark dots caused by the camera dust may become the cause of misclassified DR lesions [30,42,58].

(iii) Computational Cost: Deep neural networks are computationally rigorous, multilayered algorithms consisting of millions of parameters. Therefore, the convergence of such algorithms requires more computational power as well as running time [29,38,95]. Although there is no strict rule about how much data are required to optimally train the neural network, experiential studies recommend that tenfold training data produce an effective model. Furthermore, training high quality and a large number of images requires a powerful GPU, which is sometimes a very cost-effective issue [48].

(iv) dataset Size: Managing dataset size is also a common challenge faced by many researchers. Algorithms applied to a small dataset may not be able to accurately identify the severity grades of DR, and choosing too large data may lead to over-fitting [29,31,46,48].

(v) Interpretability: The power of the DL methods to map complex, non-linear functions makes them hard to interpret. Deep learning algorithms are like a black box in which the algorithm automatically extracts discriminative features from the images and associated grades [23,26,36]. Therefore, the specific features chosen by the algorithm are unknown. To date, understanding the features used by deep learning to perform calculations are an active area of research [49,83].

(vi) Legal Issues: Medical misconduct rules govern standards of clinical practice to show proper care to their patients. However, until now, no standard rules have been established to assign blame in contexts where the algorithm provides bad predictions and poor recommendations for treatment. The creation of such rules is an essential prerequisite for the worldwide adoption of deep learning algorithms in the medical field.

(vii) Ethical Issues: Some DR imaging data sets are publicly accessible for researchers. However, retrieving and collecting private dataset without formal agreement produces several ethical issues, particularly when the research contains sensitive information about the patient.

#### 3.3.2. Open Issues

Deep learning is a promising technique that can be used for diagnosing DR. However, researchers should pay attention while selecting the DL technique. DL models often require a large number of dataset to train the model efficiently, which are not publicly available or sometimes require permission from the hospitals or research labs to gain access. This restricts the number of researchers who can work in this field for those who are based at large academic medical centers where this dataset is available and for the most part eliminates the core deep learning community that has an essential algorithmic and theoretical background to advance the field. Moreover, used training data becomes an essential part to check the performance of the model; it is currently almost impossible to compare new approaches that were proposed in the literature with each other if the training data are not shared publicly while publishing the manuscript. Additionally, sometimes while training the DL model, there is not a clear methodology to understand how many layers should be added and which algorithm is used to find appropriate results for detecting DR automatically. Setting hyper-parameters while training the model also affects the performance of the model. It is difficult to judge which setting of the hyper-parameters to choose. Sometimes after retraining, parameters in the model become the cause of a decrease inaccuracy [39,94,96].

## 4. Discussion

This study used Deep learning techniques to detect the main five stages of DR. Taxonomy of several severity levels of DR according to the number of lesions present in the retinal fundus image is demonstrated in Figure 9. Due to the high damages of DR, the need for such diagnostic tools is a major requirement that can diagnose DR automatically with less involvement of experts. This literature review applies the systematic approach to estimate the diagnostic accuracy of DL methods for DR classification in patients with long-term diabetes. As compared to previously tested machine learning techniques, an advantage of DL includes an extensive reduction in the manpower required for extracting features by hand, as DL algorithms learn to extract features automatically. Moreover, incorporating DL algorithms might enhance the performance of the automatic diagnostic of the DR system.

We collected 39 studies to explore the use of deep learning for automatic DR-detection problems. For eight studies, reporting accuracy is above 95% [20,24,28,31,36,45,48,52]; three studies achieve accuracy above 90%. [25,27,37]; and for four studies accuracy score is above 80%. [19,21,23,26,46]. All these studies show the exceptional suggested performance rate required for detecting DR automatically and accurately. Seven studies reported specificity and sensitivity percentage variation between 90 and 98% [40,41,43,45,56,57,58,94,96].

One study achieves the highest Area Under the Curve (AUC) score of 100% [52]. All the mentioned studies reach the highest performance rate, demonstrating the potential for using DL approaches to detect DR efficiently, while keeping in mind that the included studies vary concerning target condition, the dataset used for training, and the reference standards. At some point, comparison of studies is possible between 6 studies from all 39 selected studies, as they use the same validation dataset (Kaggle) to detect five-stage severity levels of DR by using CNN [21,22,38,42,83].

From all these studies, one study [21] achieves the highest accuracy score of 80.8% on Kaggle and [42] reaches 92.2% specificity and 80.28% sensitivity at a high-sensitivity set point. The CNN model’s accuracy is considered as a strong reference standard because most of the experts expected to improve the performance and accuracy to diagnose the disease correctly as compared to detecting disease by the human grader. Nevertheless, some aspects are still changing, and it is difficult to say if the high-performance score is due to an algorithm or a combination of multiple features. Besides using the Kaggle dataset, ten studies used the Messidor-2 dataset to validate their results and achieve the highest performance scores.

DL algorithms can easily be implemented into a screening program in numerous ways. Further leading to the limitations of the primary studies, it is worth noticing that the DL model could be only validated using high-quality images and also needed a huge number of images to train the model e.g., Messidor-2, STARE, and DRIVE dataset. The images present in the dataset are not necessarily of good quality, including noise and distortion which could lead to misclassification of the model’s performance. Secondly, the majority of the involved studies used privately collected dataset that are not publicly accessible by the researchers to validate their results, so others could not validate their performance accuracy. Lastly, training a large number of images needs GPU to run an algorithm that is sometimes not very cost-effective [22,28]. Given the above-mentioned limitations, there is a need to make improvements before completely replacing the manual system with CAD tools. While our results are based on evidence-based methodology, this study still has some limitations. Firstly, the search strategy could have been more sensitive, running search string on search engines to collect studies that belong to JCR journals and CORE conferences. Moreover, performing a snow-balling approach to collect papers may have led to the inclusion of some more studies. Lastly, having incomparable studies, it was difficult to make an expectation of how the DL algorithm would perform in the real world. The purpose of this study is to provide an increased focus on the development of different DL algorithms and their performance to classify various stages of DR as the included studies show great promise.

The focus of this study was only detecting different stages of DR using deep learning techniques and not focused on lesions detection, DME, or other retinal diseases. In the future, it would be quite interesting to see whether combining DR classification with other detection techniques improves the performance of the algorithm or not. Moreover, studies should include sensitivity, AUC, specificity, confusion matrix, false positive, and false negative values, because these are important to access patient safety.

In conclusion, deep learning is currently the hottest topic (as it can be seen from the selected studies that the majority of the work has been completed in 2019) and the leading method for automatically classifying DR in patients having long-term diabetes based on diagnostic performance. Even though CAD systems cannot completely outperform humans, it is suggested to take advantage of deep learning and build semi-automated deep learning models to be used in screening programs to overcome the burden of ophthalmologists.

### Threats to Validity

This study is conducted in May 2021, so studies that appeared after that date would not have been captured. Reflecting on this methodology, this study has the following limitations.

Construct Validity: The search string is built using keywords that can extract all relevant papers, but there is a possibility that the addition of some extra keywords may alter the final results. The search string for finding results was run in IEEE explore, ACM digital library, PLOS, ResearchGate, Science direct, ArXiv, PubMed, and Springer. These digital libraries were considered a major source of data extraction in our area of interest. We did not run a research string on Google Scholar to find any related study. We believe that most of the studies to detect DR automatically can be found in these digital libraries, including all ranks of conferences and journals.

Internal validity: Internal validity deals with data analysis and the extraction process. Some studies signify overlapping contributions and areas of focus; generally, it has also been noted that one study influences and focuses only a single component of the research area. For example, improvement in the previously published study is improved by adding one or more features [22,28]. In such cases, a study that claims contribution in the area of focus has been considered for categorization. In some studies, different titles were used for the DR detection; but after reading the whole paper, those were categorized based on the DR severity stages.

External validity: Papers from various source engines were examined and selected by the authors who used the university’s access to the database to extract data, but some papers might have been ignored due to the limited access to digital libraries. This thread was managed by requesting the full article from the original authors but some authors did not reply. While shortlisting articles that were most relevant to the area of focus, some papers might be discarded due to the presence of one or more exclusion criteria. The time limit was introduced in the search for the published studies. However, the representation of the selected studies might be affected.

Conclusion validity: Threats to conclusion validity were managed by a clearer representation of each step of systematic study. While examining the study, it is put into consideration that no identification of incorrect relationships is made that may lead to incorrect conclusions. In the author’s opinion, a slight difference particular to some publication misclassification and selection bias would not change the main conclusions which were drawn from the 61 studies selected in our systematic study.

## 5. Future Directions

This study has revealed the fact that deep learning can be useful in DR detection and classification, but it still has several aspects to be open for research. It has been observed that many deep learning and advanced computation techniques have been used for the solutions of DR problems. However, due to the gap in the medical and technical field, the interpretation and accuracy of solutions concerning clinical understanding are still under discussion. Therefore, the result of this study did not include clinical accuracy and effectiveness. Another challenge is designing the deep-learning models, because these models required a huge amount of dataset. As described in the previous section, the availability of a dataset is no more a problem, rather the image annotations are one of the major issues because expert ophthalmologists are required to develop accurate annotation and thus lead the better deep-learning models. Data augmentation samples are also required for real-time DR dataset, and the development of knowledge-based data augmenting features is required for robust deep learning techniques. Moreover, image classification for DR problems requires a variety of samples for fundus images, but the available dataset are not appropriate for every kind of DR problem. Particularly, these images create a bias for DR classifications, thus this is another research direction to create morphological lesion variation and extensive data augmentation methods for preserving and classifying different kinds of DR problems. The summary of DR-related research issues and their solutions is presented in Table 14.

## 6. Conclusions

Diabetic retinopathy is the leading cause of severe eye conditions that can damage the eye retina vessels and cause vision problems. Late detection of DR problems can harm retina vessels dangerously, thus can lead to blindness. However, early detection of DR problems can prevent the potential damage. Computational methods, including image processing, machine learning, and deep learning, have been used for DR early detection. Deep learning has opened a new paradigm for designing and developing the models for the identification of DR complications including segmentation and classification. Numerous deep learning techniques have been implemented to detect different complex stages of diabetic retinopathy. In this study, we have performed a review to accurately present the state of the art in deep learning methods to detect and classify DR. Then, we have presented the taxonomy and DR grading schemes with an in-depth analysis of all the related DR problems. Moreover, we have identified major challenges and accomplishments of the studies. Eight major publication portals were used to search the relevant articles. The analysis and review have presented the main DL techniques used for multi or binary classification of DR. Further, we have provided a thorough comparison of studies based on used architecture, performance, and tools. We also have highlighted the primary data sets used by the community of researchers according to the dataset type, image type, and source. Lastly, we have provided an insight into the open issues and challenges researchers face while detecting diabetic retinopathy automatically with the help of DL methods, which also provide potential future research directions in this area. Generally, the deep learning methods have overtaken the traditional manual detection methods. This study has provided a comprehensive understanding of importance and future insights of using deep learning methods for early detection which can suggest the patients according to the severity of the problem as 35% studies used convolutional neural networks (CNNs), 26% implemented the ensemble CNN (ECNN), 13% of the studies have used Deep Neural Networks (DNN) for DR Classification. The in-depth analysis of algorithms used for DR classification also have been presented in this study. This review gives a comprehensive view of state-of-the-art deep-learning based methods related to DR diagnosis and will help researchers to conduct further research on this problem.

## Figures and Tables

**Table 1:** Comparison with other studies.

Description	[17]	[18]	[19]	This Paper
Diabetic Retinopathy Grading Protocols	✓	**X**	**X**	✓
DR Screening Detection Techniques	**X**	**X**	**X**	✓
Taxonomy of Diabetic Retinopathy (DR)	**X**	**X**	**X**	✓
Clinical Features of Retinal image for DR Detection	✓	**X**	**X**	✓
Open Issues	✓	**X**	**X**	✓
Solution to the DR based research Problems	**X**	**X**	**X**	✓
DR dataset	✓	**X**	✓	✓
Year	2019	2020	2018	2022

**Table 2:** Research Questions.

RQ No	Questions	Motivation
RQ1	What clinical features of retinal image are required for DR detection and classification, and which deep learning methods are mostly used to classify DR problems?	To answer this question, an overview of the current trends in deep learning in DR classification has been reviewed. The answer to this research question will help researchers in selecting the best deep learning technique to use as a baseline in their research.
RQ2	Which DR dataset have been acquired, managed, and classified to identify several stages of DR?	Identify available dataset to help researchers to use dataset as a benchmark and can compare the performance with their work.
RQ3	What are the open issues and challenges of using deep learning methods to detect DR?	This question will allow researchers to recognize open research challenges and future directions in order to detect DR by using more advanced deep learning techniques.

**Table 3:** Exclusion and Inclusion Criteria.

Inclusion Criteria	Exclusion Criteria
**IC1:** Studies that only focus on deep learning algorithms to detect diabetic retinopathy severity levels	**EC1:** Papers that do not focus on detecting DR by using deep learning techniques.
**IC2:** Full-text articles	**EC2:** Studies that examine only lesions e.g., micro-aneurysms, hemorrhages, exudates, cotton wool spots, etc.
**IC3:** Paper written in English language	**EC3:** Papers not published in a complete form or the form of a book, tutorial, symposium, workshop, presentation, or an essay.
	**EC4:** Papers not presented in the English language.
	**EC5:** Publication date is before the year 2015.

**Table 4:** Selection and Results.

Digital Library	No of Studies Found	No of Studies Selected
IEEE Xplore	125	9
Science Direct	53	5
PLOS One	10	1
ACM	9	1
Springer	25	5
Arxiv	12	4
Research Gate	19	9
PubMed	10	5

**Table 5:** Quality Assessment Criteria.

Criterion	Rank	Score
(a) The study provides a clear contribution to detect DR by using deep learning methods	Yes	1
	No	0
(b) The study documented the clear limitation of work while detecting DR	Yes	1
	No	0
(c) The study was methodologically explained so that it can be trusted	Yes	1
	No	0
	Partially	0.5
(d) The size of the selected dataset and the collection methods mentioned	Yes	1
	No	0
(e) Jounal/ Confence/ Symposium Ranking	Q1	2.5
	Q2	2
	Q3	1.5
	Q4 & Q5	1
	Core A	1.5
	Core B	1
	Core C	0.75
	IEEE / ACM Sponsored	0.25
	Others	0

**Table 6:** DR Detection Tools, Techniques, Methodologies, and Performance Evaluation (I).

Ref. No	DL Method	Major Focus	Environment	Performance Criteria
[14]	CNN	Propose CLEAR-DR CAD system via deep radiomic sequencer	No	Accuracy = 73.2%
[23]	CNN	Propose Siamese-like CNN architecture which accepts input as binocular fundus images	No	AUC = 95.1%, kappa score = 82.9
[24]	BNCNN	Redesign the LeNet model by adding batch normalization layer with CNN to effectively preventing gradient diffusion to improve model accuracy	No	Accuracy = 97.56%
[25]	DNN	Proposed modification of Inception-V3 model to grade four severity levels of DR	MXNET	Accuracy = 88.73%, precision = 95.77, Recall = 94.84
[26]	Ensemble CNN	Combine five models; Resnet50, Inceptionv3, Xception, Dense121, and Dense169	Keras, Tensorflow	Accuracy = 80.8%, Precision = 86.72, Recall = 51.5, F-score = 63.85
[27]	Ensemble CNN	Combine three models: inceptionv3, Xception, and inceptionResNetV2	Keras	Accuracy = 97.15%, Precision = 0.96, Recall = 0.96, F1-score = 0.96
[28]	WP-CNN	Build various weighted path CNN networks and optimized by backpropagation. WP-CNN105 achieves the highest accuracy.	No	Accuracy = 94.23%, F1-score = 0.9087
[29]	Ensemble CNN	Five ensemble models VGG-16, ResNet-18, SE-BN-inception, GoogleNet, and DenseNet were used as benchmark for DR grading	Caffe	Accuracy = 82.84%
[30]	OCTD-Net	Develop novel deep network OCTD-NET. Consist of two features one for feature extraction and other for retinal layer information	Keras	Accuracy = 92%
[31]	GoogLeNet	Propose modification of GoogLeNet convolutional neural network	No	Accuracy = 98%
[32]	Ensemble CNN	Ensemble CNN VGG net and ResNet models used as ensemble	No	AUC = 97.3%
[33]	Deep Multi-Instance Learning	Image patches extracted from the preprocessing step regularly and then fed into CNN based patch level classifier	MatConvNet	Precision = 86.3, F1-score = 92.1
[34]	Fully connected Network	Construct U-Net based regional segmentation and diagnosis model	Keras	PM coefficient is 2.55% lower
[35]	DCNN	Transfer learning used for initial weight initialization and for feature extraction	No	Accuracy = 93.6%, 95.8%
[36]	DR-Net	Develop DR-Net framework by fully stacked convolution network to reduce overfitting and to improve performance	imageMagick, OpenCV	Accuracy = 81.0%
[37]	Ensemble CNN	Three models, inception V3, Resnet152, and inception-Resnet-v3 put together that work individually and Adaboost algorithm is used to merge them.	Ubuntu	Accuracy = 88.21%
[38]	DNN	Neural network with 28 convolutional layers, after each layer batch normalization and ReLu applied except the last one Network trained with inception-v3 model	Tensorflow, Android studio	Accuracy = 73.3%
[39]	CNN	Network consists of range of convolutional layers that converts pixel intensities to local features before converting them to global features	No	Accuracy = 97.8%
[40]	CNN	Propose CNN model with the addition of regression activation map	No	Accuracy = 94%, 80%

**Table 7:** DR Detection Tools, Techniques, Methodologies, and Performance Evaluation (II).

Ref. No	DL Method	Major Focus	Environment	Performance Criteria
[41]	Graph-NN	Propose GNN model which consists of two features. One is to extract region-of-interest focusing only regions to remove noise while preprocessing and others in applying GNN for classification	No	Accuracy = 80%
[42]	CNN	Constructed a model in which artificial neurons are organized in a hierarchical manner which are able to learn multiple level of abstraction	No	Accuracy = 79.3%
[43]	Deep CNN	Use VGG-16 DCNN to automatically detect local features and to generate a classification model	No	Specificity 97%, Sensitivity 96.7%
[44]	CNN	Demonstrates the potential of CNN to classify DR fundus images based of severity in real times	No	AUC 96.6%, Specificity = 97.2 %, Sensitivity = 94.7%
[45]	Ensemble DNN	Build high quality medical imaging dataset of DR also propose a grading and identification system called DeepDR and evaluate the model using nine validity matrices	No	Specificity = 92.29%, Sensitivity = 80.28%
[46]	Modified Hopfield NN	Propose Modify Hopfield neural network to handling drawbacks of conventional HNN where weigh values changed based on the output values in order to avoid the local minima	Keras	AUC = 90.1 %, Sensitivity = 84.6, 90.6, Specificity = 79.9, 90 %
[47]	Deep CNN	DCNN pooling layer is replaced with fractional max pooling to drive more discriminative features for classification also use SVM to classify underlying boundary of distribution. Furthermore build an app called Deep Retina	No	Accuracy = 99.25%, Specificity = 99.0%
[48]	DCNN	Data driven features learned from deep learning network through dataset and then these deep features were propagated into a tree based classification model that output a final diagnostic disease	No	Accuracy = 86.71, Specificity = 90.89, Sensitivity = 89.30
[49]	DNN	Propose Alex Net DNN with caffeNet model to extract multi-dimensional features at fully connected DNN layers and use SVM for optimal five class DR classification	No	AUC = 97%, Sensitivity = 94, Specificity = 98
[50]	CNN	Build an automated system to detect DR IDX-DR X2.1 composed of client software and analysis software. The device applied a set of CNN based detectors to examine each image	No	Accuracy = 97.93
[51]	DCNN	Proposed a systematic computation model using DCNN for DR classification and assessed performance on non-open dataset and found that model achieves better results with only a small fraction of training set images	No	AUC = 98.0%, Sensitivity = 96.8, Specificity = 87.0
[52]	CNN	Employ LSTM, CNN, and their combination for extracting complex features to input into heart rate variability dataset.	No	Sensitivity = 88.3, Specificity = 98.0

**Table 8:** DR Detection Tools, Techniques, Methodologies, and Performance Evaluation (III).

Ref. No	DL Method	Major Focus	Environment	Performance Criteria
[53]	CNN	Build a network using CNN and data augmentation to identify the intricate features like Micro-aneurysms, exudates, cotton wool spots to automatically diagnosis DR without user input	No	Accuracy= 95.7%
[54]	Deep Densely Connected NN	Pioneer work to use densely connected NN to classify DR with the motivation behind to deploy network with more deep supervision to extract comprehensive features from the images.	Scikit-learn	Accuracy= 75, Sensitivity= 95, AUC = 100, Precision = 0.95, Recall= 0.98, F1-score = 0.97, Specificity = 0.98
[55]	CNN	Adopted CNN-independent adaptive kernel visualization technique to validate deep learning model for the detection of referable diabetic retinopathy	Keras	AUC= 0.93, Specificity = 91.6, Sensitivity = 90.5
[56]	CNN	Deploy CNN to evaluate the performance of deep learning system in detecting RDR by using 10 different dataset	No	AUC = 0.924, Sensitivity = 92.180, Specificity = 94.50
[57]	Deep Visual Features	Propose a novel method to detect SLDR without performing pre and post processing steps on retinal images through learning of deep visual features and gradient location orientation histogram	Matlab	AUC = 0.924, Sensitivity = 92.180, Specificity = 94.50
[58]	CNN	Use CNN model that uses a function that combine nearby pixels into local features and then combined it into global features. The algorithm does not explicitly detect lesions but recognize them using local features	No	AUC = 97.4, Sensitivity = 97.5, specificity = 93.4
[59]	Tuning Inception-v4 principal component analysis (PCA)	Comparison of different CNN algorithms to test the extensive DR image classification .	No	Accuracy= 99.49, Sensitivity= 98.83, Specificity = 99.68
[60]	Principal Component Analysis (PCA) DNN-PCAFirefly	Performed DR and NDPR dataset analysis for early detection of DR to prevent the damages.	No	Accuracy= 97, Sensitivity= 96, Specificity = 96
[61]	Synergic Deep Learning (SDL)	Prepare a classifier and model as SDL for fundus DR detection.	No	Accuracy= 99.28, Sensitivity= 98, Specificity = 99
[62]	Lesion-aware Deep Learning System (RetinalNET)	Developed a model and classifier for prediction of end stage DR in Chinese patients .	No	HR 2.18, 95 confidence interval (CI) 1.05–4.53, P=0.04
[63]	Ensemble DCNN	Prepared a classifier for detection of DR by using fundus images.	No	Accuracy= 99.28, Sensitivity=98
[64]	Patch-based CNN	Use filters for fundus dataset pre-processing to train patch based CNN classifier.	No	Accuracy= 95.35, Sensitivity=97
[65]	FCN VGG-16	Developed the deep learning FCN VGG 16 Trainer for understanding of Fundus images for early detection of DR.	No	RPR=0.822, RPR=0.831
[66]	Patch Based CNN/PCA	Prepare a Deep learning patch based CNN/PCA classifier for vessel tracking in DRIVE dataset for DR image learning.	No	Accuracy= 0.9701

**Table 9:** DR Detection Tools, Techniques, Methodologies, and Performance Evaluation (IV).

Ref. No	DL Method	Major Focus	Environment	Performance Criteria
[67]	Patch Based FCN	Segmented the DR vessel data by patch based FCN for Deep DR prediction	No	SN = 76.91, SP = 98.01, AUC = 0.974, ACC = 95.33
[68]	FCN/CRF	Biomedical image processing by FCN and CRF deep learning models to train and test the DRIVE and STARE dataset.	No	SN = 72.94, ACC = 94.70, SN = 71.40, ACC = 95.45
[69]	Multi-level FCN	Used DRIVE, STARE, and CHASE dataset for deep segmentation of retinal vessels also prepared comparison of the optimization model of these three dataset.	No	DRIVE[SN = 77.79, SP = 97.80, AUC = 0.9782], STARE[SN = 95.21, SP = 81.47, AUC = 98.44] CHASE[SN = 96.76, SP = 76.61, AUC = 98.16]
[70]	Patch-based DSAE	Deep learning based ensembling of classifier was used to achieve label-free DR angiography for efficient retinal classification and segmentation.	No	Accuracy = 95.3
[71]	Patch-based SDAE	Used deep learning to understand the segmentation of retinal vessel in DR on DRIVE, CHASE, and STARE dataset.	No	SN = 75.6, SP = 98, AUC = 0.9738 ACC = 95.27
[72]	Deep CNN	Deep Learning based PMNPDR diagnostic system has been proposed for non-proliferative DR	No	Sensitivity dark lesions = 97.4, 98.4 and 95.1, Sensitivity bright lesions = 96.8, 97.1 and 95.3
[10]	Deep CNN	Prepared a human-centric evaluation on the dataset of several clinics to detect the DR by using deep learning. This dataset has been retrieved from the systems deployed in the clinic.	No	Sensitivity>90
[73]	CNN-based AlexNet, GoogLeNet, and ResNet50	Introduced smartphone diagnostic system for detecting the DR and used Deep Learning classifiers frameworks.	No	accuracy of 98.6, sensitivity and a 99.1
[74]	Inception-v4	Detection of early DR symptoms and severity by recognizing features.	No	accuracy of 96.11, Kappa Score 89.81
[75]	VGG16, DenseNet121	Prepared a model based on high resolution images to classify and early detection.	No	accuracy of 96.11, Kappa Score 89.81
[76]	CNN	Proposed the architecture by using nonlinear ReLU function and batch normalization by CNN.	No	accuracy of 98.7, sensitivity 0.996
[77]	CNN	Proposed a method to join to DR and DME by using CANet	No	accuracy of 65.1
[78]	DCNN	Prepared an architecture using DCCN via gated attention for classification of DR images.	No	accuracy 82.54, Kappa score 79
[79]	VeriSee	Develop a deep learning based assessment software to validate the DR severity.	No	accuracy 89.2, Specificity 90.1 and, AUC 0.95
[80]	RFCN, SDD-515, VGG16	Prepared a deep learning based model for enhancing the small object detection for better classification.	No	accuracy 98, Specificity 99.39 and, percision 92.15
[81]	ResNet50, EfficientNet-b0, Se-ResNet50	Proposed a framework to train the fundus images future prediction of lesions and other DR issues.	No	accuracy 94, Specificity 95 and, Senstivity 92

**Table 10:** Quality Assessment Results (I).

Ref. No	Study Type	Dataset	GPU	Scoring					Total
				(a)	(b)	(c)	(d)	(e)	
[14]	Experiment	Kaggle	No	1	0	0.5	1	2.5	5
[23]	Experiment	Kaggle	NVIDIA GeForce	1	1	1	1	2.5	6.5
[24]	Experiment	Collect 6 billion records from 301 hospitals	No	1	1	1	1	2.5	6.5
[25]	Experiment	Collect 447 images from three different \clinical departments	2 Tesla K40 GPUs and 64G of RAM	1	1	1	1	2.5	6.5
[26]	Experiment	Kaggle	NVIDIA Tesla K40	1	0	1	1	2.5	5.5
[27]	Experiment	Nine medical records were used	NVIDIA Tesla K40 GPU and 64 GB RAM	1	1	1	1	2.5	6.5
[28]	Experiment	STARE	No	1	0	0.5	1	2.5	5
[29]	Experiment	Fundus image dataset collected from Chinese population	NVIDIA Tesla k40	1	0	0.5	1	2.5	5
[30]	Experiment	OCT images provided by Wenzhou Medical university	GeForce GTX Titan X and 12 GB RAM	1	0	1	1	2.5	5.5
[31]	Experiment	Collect 9939 posterior photos from jichi Medical University	Titan X with 12 GB RAM	1	0	0.5	1	2.5	5
[32]	Case study	76,370 fundus images from Singapore integrated DR program	No	1	0	0.5	1	2	4.5
[33]	Experiment	Kaggle, Messidor, DIARETDR1	4 NVIDIA GeForce Titan X	1	1	1	1	2	6
[34]	Experiment	No	556 images from china west hospital	1	1	0.5	0	2	4.5
[35]	Experiment	Obtained from SiDRP	No	1	0	0	0	1	2
[36]	Experiment	Kaggle, Drive, Messidor	NVIDIA Tesla k20	1	0	0	1	1.5	3.5
[37]	Experiment	30,244 fundus images from Beijing Tongren Eye center	NVIDIA Tesla P40 of 24 GB RAM	1	0	0.5	0	0.25	1.75
[38]	Model	16,789 fundus images	No	1	0	0.5	0	0.25	1.75
[39]	Experiment	25,326 fundus images from screening program in Thailand	No	1	0	1	1	1.5	4.5
[40]	Case Study	Messidor 2	No	1	1	1	1	1.5	5.5
[41]	Experiment	Kaggle	No	1	0	1	1	1.5	4.5
[42]	Experiment	IDRiD	Tesla p100	1	0	1	1	1.5	4.5
[43]	Experiment	EyePACS, Messidor-2, 19,230 images from DM population	No	1	1	1	1	2.5	6.5
[44]	Experiment	Obtained 132 images from Saneikai Tsukazaki Hospital and Tokushima University Hospital	No	1	1	1	1	2	6
[45]	Experiment	Kaggle	NVIDIA GTX980Ti, mazon EC2 instance containing NVIDIA	1	0	1	1	1.5	4.5
[46]	Experiment	13,767 images obtained from ophthalmology, endocrinology and physical examination centers	NVIDIA TeslaK40	1	1	1	1	2.5	6.5
[47]	Experiment	Modified Hopfield NN	No	1	0	0.5	1	2.5	5

**Table 11:** Quality Assessment Results (II).

Ref. No	Study Type	Dataset	GPU	Scoring					Total
				(a)	(b)	(c)	(d)	(e)	
[48]	Experiment	Kaggle	Intel dual core processor with 4 GB RAM	1	0	0.5	1	2	4.5
[49]	Experiment	Messidor-2, EyePACS, E-Ophtha	No	1	1	1	1	1.5	5.5
[50]	Experiment	Kaggle	Intel dual core processor, iphone 5	1	1	1	1	2.5	6.5
[51]	Experiment	Obtained from EyeCheck project and university of lowa, Messidor-2	No	1	1	1	1	1.5	5.5
[52]	Experiment	Privately collected Data	No	1	1	1	1	2.5	6.5
[53]	Experiment	41122 colour DR images from screening process in finland	No	1	1	1	1	2.5	6.5
[54]	Experiment	Collect ECG of 20 people	No	1	0	0.5	1	2	4.5
[55]	Experiment	Kaggle	NVIDIA k40c	1	1	1	1	1	5
[56]	Experiment	66,790 images collected from Label database in China	No	1	1	0.5	1	2	5.5
[57]	Experiment	Half a million images were collected from 10 different private locations	No	1	1	1	1	2.5	6.5
[58]	Experiment	Messidor, DIARETDR, retinopathies were collected from HUPM Spain	No	1	1	1	1	2	6
[59]	Experiment	eyePACS, Messidor-2	No	1	1	1	1	2.5	6.5
[60]	Experiment	MESSIDOR	TI-V4(PCA)	1	1	1	1	2.5	6.5
[61]	Experiment	Diabetic Retinopathy Debrecen dataset from UCI machine learning repository	No	1	1	1	1	2.5	6.5
[62]	Experiment	Messidor	No	1	1	1	1	2.5	6.5
[63]	Experiment	eyePACS, Messidor-2	No	1	1	1	1	2.5	6.5
[64]	Experiment	DRIVE	No	1	1	1	1	2.5	6.5
[65]	Experiment	DRIVE, STARE and CHASE	No	1	1	1	1	2.5	6.5
[66]	Experiment	DRIVE, STARE	No	1	1	1	1	2.5	6.5
[67]	Experiment	DRIVE	No	1	1	1	1	2.5	6.5
[68]	Experiment	DRIVE	No	1	1	1	1	2.5	6.5
[69]	Experiment	DRIVE, STARE	No	1	1	1	1	2.5	6.5
[70]	Experiment	eyePACS, Messidor-2	No	1	1	1	1	2.5	6.5
[71]	Experiment	DRIVE	No	1	1	1	1	2.5	6.5
[72]	Experiment	eyePACS, Messidor-2	No	1	1	1	1	2.5	6.5
[10]	Experiment	DRIVE, STARE and CHASE	No	1	1	1	1	2.5	6.5
[73]	Case study	real time-Thiland	No	1	1	1	1	2.5	6.5
[74]	Experiment	EyePACS, Messidor, Messidor-2, IDRiD,UoA-DR	No	1	1	1	1	2.5	6.5
[75]	Experiment	207,130 retinal images	No	1	0	1	1	2.5	5.5
[76]	Experiment	3662 retinal images	No	1	0	0.5	0	0	1.5
[77]	Experiment	89 retinal images	No	1	0	1	0	2	4
[78]	Experiment	IDRiD, Messidor	No	1	1	1	1	2.5	6.5
[79]	Experiment	Kaggle	No	1	0	1	1	2.5	5.5
[80]	Experiment	EyePACS	No	1	0	0.5	1	2.5	5
[81]	Experiment	Messidor	No	1	0	1	1	2	5
[82]	Experiment	Kangbuk Samsung Hospital dataset	No	1	0	0.5	1	2.5	5

**Table 12:** Diabetic Retinopathy Grading Protocols.

Grading Protocol	Rank
American Academy of Ophthalmology (AAO) & International Clinical Diabetic Retinopathy (ICDR)	No DR, Very Mild, Mild, Moderate, Severe, Very Severe
Early Treatment of Diabetic Retinopathy Study (ETDRS)	0-Level 10
	1-Level 20
	2-Level 35, 43, 47
	3-Level 53
	4-Level 61, 65, 71, 75, 81, 85
Scottish DR Grading	R0-DR
	R1-mild NPDR
	R2 &R3-pre-Moderate and Severe NPDR
	R4- PDR
National Screening Committee—UK	R0-DR
	R1-mild NPDR
	R2-Moderate and Severe PDR
	R3-PDR Pre-retinal fibrosis

**Table 13:** Publically available dataset to Detect DR.

Dataset	No. of Images	Format	Provided By
DRIVE	40	JPEG	Screening Program in Netherlands
Download URL: https://drive.grand-challenge.org/Download/ (accessed on 14 December 2021)			
KAGGLE	80,000	JPEG	EYEPACS
Download URL: http://Kaggle.diabetic-retinopathy-detection/data (accessed on 14 December 2021)			
DIARETDB 0 & 1	89	GT:PNG	ImageRet Project
Download URL: http://www2.it.lut.fi/project/imageret/diaretdb1/#DATA (accessed on 14 December 2021)			
MESSIDOR	1200	TIFF	Messidor Program Partner
Download URL: http://www.adcis.net/en/third-party/messidor/ (accessed on 14 December 2021)			
STARE	402	PPM	Shiley Eye Centre
Download URL: http://cecas.clemson.edu (accessed on 14 December 2021)			
AREDS	72,000	JPEG	National Eye Institute (NEI)
Download URL: https://www.ncbi.nlm.nih.gov/gap/ (accessed on 14 December 2021)			
EyePACS-1	9963	JPEG	U.S. screening program
Download URL: http://www.eyepacs.com/data-analysis/ (accessed on 14 December 2021)			
e-ophtha	463	JPEG, PNG	French Research Agency (ANR)
Download URL: https://www.adcis.net/en/third-party/e-ophtha/ (accessed on 14 December 2021)			
ORIGA	625	BMP	Institute for Infocomm Research, A*STAR, Singapore
Download URL: available free on request			
SCES	1676	BMP	Singapore Eye Research Institute
Download URL:http://biomisa.org/index.php/glaucoma-database/ (accessed on 14 December 2021)			
SIDRP 2014–2015	71,896	BMP	Singapore National DR Screening Program, Singapore
Download URL:http://messidor.crihan.fr/index-en.php (accessed on 14 December 2021)			

**Table 14:** Research Gaps and Future directions.

Research Gap & Issue	Description	Solution
Clinical Results	Ophthalmologists feedback is required in order to check the accuracy of the deep learning predictor.	Cross-Database Validation
Data Augmentation	Accurate data augmentation is an expensive solution and expert Ophthalmologist services are required in every angle of lesion image.	Generative Adversarial Networks (GANs), New data augmentation techniques with fewer learnable parameters
Class Imbalance	The number of DR cases is much lower than normal cases	Data Augmentation Techniques, Geometric Transformations
Lack of Uniformity	Angle of images are not uniform, out of focus, and causes the diffusion of light in the retina	Generative Adversarial Networks (GAN) New Augmentation Techniques
Translation Effect	Variability and screening programs do not follow a standard and cause issues	Translation Standards are required
Race Scaling	It has been observed that darker retina vascular properties are comparatively different to the light tone retina	Heterogeneous cohorts parameters

## Data Availability

Not Applicable.

## Abbreviations

BDR	Background diabetic retinopathy
DMI	Diabetic macular ischemia
PDR	Proliferative diabetic retinopathy
NPDR	Non-Proliferative Diabetic Retinopathy
CVF	Central Visual Field
IRMA	Intraretinal Microvascular Abnormalities
BC	Base Curve
MA	Microaneurysm
DM	Mellitus
R2	Pre-proliferative Retinopathy
DME	Diabetic Macular Edema
ETDRS	Early Treatment Diabetic Retinopathy Study
HMs	Hemorrhages
EXs	Exudates
HEs	Hard Exudates
SEs	Soft Exudates
VB	Venous Beading
NV	Neovascularization
ME	Macular Edema
OD	Optic Disc
AMD	Age-Related Macular Degeneration
AAO	American academy of Ophthalmology
ICDR	International Clinical Diabetic Retinopathy

## Appendix A

**Table A1:** Data Extraction Strategy.

DE ID	Data Extraction	Description	Type
DE1	Study Identifier	Unique Identity for every study	General
DE2	Publication Type	Nature of Publication (Journal/Conference/Symposium etc.)	General
DE3	Bibliographic References	Title, Author Name, Publication Year, Location	General
DE4	Dataset	Type of Dataset, Total number of images in each dataset, Source	RQ2
DE5	Deep Learning Architecture	Type of deep learning methods used in selected studies	RQ1
DE6	Type of Classification	Multi-class or Binary	RQ2

## References

[1] (2019). Diabetes, Heart Disease, and Stroke. https://www.niddk.nih.gov/health-information/diabetes/overview/preventing-problems/heart-disease-stroke.

[2] ‘Diabetic Retinopathy-Symptoms and Causes’, Mayo Clinic. https://www.mayoclinic.org/diseases-conditions/diabetic-retinopathy/symptoms-causes/syc-20371611.

[3] Introduction to Diabetes and Diabetic Retinopathy. https://www.visionaware.org/info/your-eye-condition/diabetic-retinopathy/1.

[4] Quellec G., Lamard M., Josselin P.M., Cazuguel G., Cochener B., Roux C. (2008). Optimal wavelet transform for the detection of microaneurysms in retina photographs. IEEE Trans. Med Imaging.

[5] Abràmoff M.D., Folk J.C., Han D.P., Walker J.D., Williams D.F., Russell S.R., Massin P., Cochener B., Gain P., Tang L. (2013). Automated Analysis of Retinal Images for Detection of Referable Diabetic Retinopathy. JAMA Ophthalmol..

[6] Wilkinson C.P., Ferris F.L., Klein R.E., Lee P.P., Agardh C.D., Davis M., Dills D., Kampik A., Pararajasegaram R., Verdaguer J.T. (2003). Proposed international clinical diabetic retinopathy and diabetic macular edema disease severity scales. Ophthalmology.

[7] Wong T.Y., Cheung C.M.G., Larsen M., Sharma S., Simó R. (2016). Diabetic retinopathy. Nat. Rev. Dis. Prim..

[8] (2010). EyeRound.org. https://webeye.ophth.uiowa.edu/eyeforum/tutorials/Diabetic-Retinopathy-Med-Students/Classification.htm.

[9] Shilpa J., Karule P.T. (2018). A review on exudates detection methods for diabetic retinopathy. Biomed. Pharmacother..

[10] Beede E., Baylor E., Hersch F., Iurchenko A., Wilcox L., Ruamviboonsuk P., Vardoulakis L.M. A human-centered evaluation of a deep learning system deployed in clinics for the detection of diabetic retinopathy. Proceedings of the 2020 CHI Conference on Human Factors in Computing Systems.

[11] American Academy of Ophthalmology. https://www.aao.org/eyenet/article/vitreous-hemorrhage-diagnosis-treatment-2.

[12] Brown G.C., Brown M.M., Hiller T.Y.R.I.E., Fischer D.A.V.I.D., Benson W.E., Magargal L.E. (1985). Cotton-wool spots. Retina.

[13] Difference Between Machine Learning And Deep Learning. http://www.iamwire.com/2017/11/difference-between-machine-learning-and-deep-learning/169100.

[14] Asiri N., Hussain M., Abualsamh H.A. (2018). Deep Learning based Computer-Aided Diagnosis Systems for Diabetic Retinopathy: A Survey. arXiv.

[15] Zhao L., Ren H., Zhang J., Cao Y., Wang Y., Meng D., Li L. (2020). Diabetic retinopathy, classified using the lesion-aware deep learning system, predicts diabetic end-stage renal disease in chinese patients. Endocr. Pract..

[16] Mansour R.F. (2017). Evolutionary computing enriched computer-aided diagnosis system for diabetic retinopathy: A survey. IEEE Rev. Biomed. Eng..

[17] (2019). National Eye Institute. https://www.nei.nih.gov/learn-about-eye-health/eye-conditions-and-diseases/diabetic-retinopathy.

[18] Pires R., Avila S., Wainer J., Valle E., Abramoff M.D., Rocha A. (2019). A data-driven approach to referable diabetic retinopathy detection. Artif. Intell. Med..

[19] Nielsen K.B., Lautrup M.L., Andersen J.K., Savarimuthu T.R., Grauslund J. (2019). Deep learning–based algorithms in screening of diabetic retinopathy: A systematic review of diagnostic performance. Ophthalmol. Retin..

[20] Alyoubi W.L., Shalash W.M., Abulkhair M.F. (2020). Diabetic retinopathy detection through deep learning techniques: A review. Inform. Med. Unlocked.

[21] Chler J., Hopewell S. (2013). Cochrane methods-twenty years experience in developing systematic review methods. Syst. Rev..

[22] Ouhbi S., Idri A., Fernández-Alemán J.L., Toval A. (2015). Requirements engineering education: A systematic mapping study. Requir. Eng..

[23] Zeng X., Chen H., Luo Y., Ye W. (2019). Automated Diabetic Retinopathy Detection Based on Binocular Siamese-Like Convolutional Neural Network. IEEE Access.

[24] Sun Y. (2019). The Neural Network of One-Dimensional Convolution-An Example of the Diagnosis of Diabetic Retinopathy. IEEE Access.

[25] Gao Z., Li J., Guo J., Chen Y., Yi Z., Zhong J. (2018). Diagnosis of Diabetic Retinopathy Using Deep Neural Networks. IEEE Access.

[26] Qummar S., Khan F.G., Shah S., Khan A., Shamshirb S., Rehman Z.U., Jadoon W. (2019). A Deep Learning Ensemble Approach for Diabetic Retinopathy Detection. IEEE Access.

[27] Zhang W., Zhong J., Yang S., Gao Z., Hu J., Chen Y., Yi Z. (2019). Automated identification and grading system of diabetic retinopathy using deep neural networks. Knowl.-Based Syst..

[28] Liu Y.P., Li Z., Xu C., Li J., Liang R. (2019). Referable diabetic retinopathy identification from eye fundus images with weighted path for convolutional neural network. Artif. Intell. Med..

[29] Li T., Gao Y., Wang K., Guo S., Liu H., Kang H. (2019). Diagnostic Assessment of Deep Learning Algorithms for Diabetic Retinopathy Screening. Inf. Sci..

[30] Li X., Shen L., Shen M., Tan F., Qiu C.S. (2019). Deep learning based early stage diabetic retinopathy detection using optical coherence tomography. Neurocomputing.

[31] Takahashi H., Tampo H., Arai Y., Inoue Y., Kawashima H. (2017). Applying artificial intelligence to disease staging: Deep learning for improved staging of diabetic retinopathy. PLoS ONE.

[32] Bellemo V., Lim Z.W., Lim G., Nguyen Q.D., Xie Y., Yip M.Y., Lee M.L. (2019). Artificial intelligence using deep learning to screen for referable and vision-threatening diabetic retinopathy in Africa: A clinical validation study. Lancet Digit. Health.

[33] Zhou L., Zhao Y., Yang J., Yu Q., Xu X. (2017). Deep multiple instance learning for automatic detection of diabetic retinopathy in retinal images. IET Image Process..

[34] Li Q., Fan S., Chen C. (2019). An Intelligent Segmentation and Diagnosis Method for Diabetic Retinopathy Based on Improved U-NET Network. J. Med. Syst..

[35] Yip M.Y.T., Lim Z.W., Lim G., Quang N.D., Hamzah H., Ho J., Hsu W. (2018). Enhanced Detection of Referable Diabetic Retinopathy via DCNNs and Transfer Learning. Proceedings of the Asian Conference on Computer Vision.

[36] Chakravarthy S.N., Singhal H., RP N.Y. DR-NET: A Stacked Convolutional Classifier Framework for Detection of Diabetic Retinopathy. Proceedings of the 2019 International Joint Conference on Neural Networks (IJCNN).

[37] Jiang H., Yang K., Gao M., Zhang D., Ma H., Qian W. An Interpretable Ensemble Deep Learning Model for Diabetic Retinopathy Disease Classification. Proceedings of the 2019 41st Annual International Conference of the IEEE Engineering in Medicine and Biology Society (EMBC).

[38] Suriyal S., Druzgalski C., Gautam K. Mobile assisted diabetic retinopathy detection using deep neural network. Proceedings of the 2018 Global Medical Engineering Physics Exchanges/Pan American Health Care Exchanges (GMEPE/PAHCE).

[39] Raumviboonsuk P., Krause J., Chotcomwongse P., Sayres R., Raman R., Widner K., Silpa-Acha S. (2018). Deep Learning vs. Human Graders for Classifying Severity Levels of Diabetic Retinopathy in a Real-World Nationwide Screening Program. arXiv.

[40] Voets M., Møllersen K., Bongo L.A. (2018). Replication study: Development and validation of deep learning algorithm for detection of diabetic retinopathy in retinal fundus photographs. arXiv.

[41] Sakaguchi A., Wu R., Kamata S.I. Fundus Image Classification for Diabetic Retinopathy Using Disease Severity Grading. Proceedings of the 2019 9th International Conference on Biomedical Engineering and Technology.

[42] Romero-Aroca P., Verges-Puig R., de la Torre J., Valls A., Relaño-Barambio N., Puig D., Baget-Bernaldiz M. (2019). Validation of a Deep Learning Algorithm for Diabetic Retinopathy. Telemed. e-Health.

[43] Nagasawa T., Tabuchi H., Masumoto H., Enno H., Niki M., Ohara Z., Mitamura Y. (2019). Accuracy of ultrawide-field fundus ophthalmoscopy-assisted deep learning for detecting treatment-naïve proliferative diabetic retinopathy. Int. Ophthalmol..

[44] Raju M., Pagidimarri V., Barreto R., Kadam A., Kasivajjala V., Aswath A. (2017). Development of a deep learning algorithm for automatic diagnosis of diabetic retinopathy. MEDINFO 2017: Precision Healthcare through Informatics.

[45] Ramach R.N., Hong S.C., Sime M.J., Wilson G.A. (2018). Diabetic retinopathy screening using deep neural network. Clin. Exp. Ophthalmol..

[46] Hemanth D.J., Anitha J., Mittal M. (2018). Diabetic retinopathy diagnosis from retinal images using modified hopfield neural network. J. Med Syst..

[47] Li Y.-H., Yeh N.-N., Chen S.-J., Chung Y.-C. (2019). Computer-Assisted Diagnosis for Diabetic Retinopathy Based on Fundus Images Using Deep Convolutional Neural Network. Mob. Inf. Syst..

[48] Gargeya R., Leng T. (2017). Automated identification of diabetic retinopathy using deep learning. Ophthalmology.

[49] Mansour R.F. (2018). Deep-learning-based automatic computer-aided diagnosis system for diabetic retinopathy. Biomed. Eng. Lett..

[50] Abràmoff M.D., Lou Y., Erginay A., Clarida W., Amelon R., Folk J.C., Niemeijer M. (2016). Improved automated detection of diabetic retinopathy on a publicly available dataset through integration of deep learning. Investig. Ophthalmol. Vis. Sci..

[51] Sahlsten J., Jaskari J., Kivinen J., Turunen L., Jaanio E., Hietala K., Kaski K. (2019). Deep Learning Fundus Image Analysis for Diabetic Retinopathy and Macular Edema Grading. arXiv.

[52] Swapna G., Vinayakumar R., Soman K.P. (2018). Diabetes detection using deep learning algorithms. ICT Express.

[53] Pratt H., Coenen F., Broadbent D.M., Harding S.P., Zheng Y. (2016). Convolutional neural networks for diabetic retinopathy. Procedia Comput. Sci..

[54] Riaz H., Park J., Choi H., Kim H., Kim J. (2020). Deep and Densely Connected Networks for Classification of Diabetic Retinopathy. Diagnostics.

[55] Keel S., Wu J., Lee P.Y., Scheetz J., He M. (2019). Visualizing Deep Learning Models for the Detection of Referable Diabetic Retinopathy and Glaucoma. JAMA Ophthalmol..

[56] Ting D.S.W., Cheung C.Y.L., Lim G., Tan G.S.W., Quang N.D., Gan A., Wong E.Y.M. (2017). Development and validation of a deep learning system for diabetic retinopathy and related eye diseases using retinal images from multiethnic populations with diabetes. JAMA.

[57] Abbas Q., Fondon I., Sarmiento A., Jiménez S., Alemany P. (2017). Automatic recognition of severity level for diagnosis of diabetic retinopathy using deep visual features. Med Biol. Eng. Comput..

[58] Gulshan V., Peng L., Coram M., Stumpe M.C., Wu D., Narayanaswamy A., Kim R. (2016). Development and validation of a deep learning algorithm for detection of diabetic retinopathy in retinal fundus photographs. JAMA.

[59] Shankar K., Zhang Y., Liu Y., Wu L., Chen C.H. (2020). Hyperparameter tuning deep learning for diabetic retinopathy fundus image classification. IEEE Access.

[60] Gadekallu T.R., Khare N., Bhattacharya S., Singh S., Maddikunta P.K.R., Ra I.H., Alazab M. (2020). Early detection of diabetic retinopathy using PCA-firefly based deep learning model. Electronics.

[61] Tymchenko B., Marchenko P., Spodarets D. (2020). Deep Learning Approach to Diabetic Retinopathy Detection. arXiv.

[62] Thakur N., Juneja M. (2018). Survey on segmentation and classification approaches of optic cup and optic disc for diagnosis of glaucoma. Biomed. Signal Process. Control.

[63] Maji D., Santara A., Mitra P., Sheet D. (2016). Ensemble of deep convolutional neural networks for learning to detect retinal vessels in fundus images. arXiv.

[64] Liskowski P., Krawiec K. (2016). Segmenting retinal blood vessels with deep neural networks. IEEE Trans. Med Imaging.

[65] Maninis K.K., Pont-Tuset J., Arbeláez P., Gool L.V. (2016). Deep retinal image understanding. Proceedings of the International Conference on Medical Image Computing and Computer-Assisted Intervention.

[66] Wu A., Xu Z., Gao M., Buty M., Mollura D.J. Deep vessel tracking: A generalized probabilistic approach via deep learning. Proceedings of the 2016 IEEE 13th International Symposium on Biomedical Imaging (ISBI).

[67] Dasgupta A., Singh S. A fully convolutional neural network based structured prediction approach towards the retinal vessel segmentation. Proceedings of the 2017 IEEE 14th International Symposium on Biomedical Imaging (ISBI 2017).

[68] Fu H., Xu Y., Wong D.W.K., Liu J. Retinal vessel segmentation via deep learning network and fully-connected conditional random fields. Proceedings of the 2016 IEEE 13th International Symposium on Biomedical Imaging (ISBI).

[69] Mo J., Zhang L. (2017). Multi-level deep supervised networks for retinal vessel segmentation. Int. J. Comput. Assist. Radiol. Surg..

[70] Lahiri A., Roy A.G., Sheet D., Biswas P.K. Deep neural ensemble for retinal vessel segmentation in fundus images towards achieving label-free angiography. Proceedings of the 2016 IEEE 38th Annual International Conference of the Engineering in Medicine and Biology Society (EMBC).

[71] Li Q., Feng B., Xie L., Liang P., Zhang H., Wang T. (2016). A cross-modality learning approach for vessel segmentation in retinal images. IEEE Trans. Med Imaging.

[72] Qiao L., Zhu Y., Zhou H. (2020). Diabetic retinopathy detection using prognosis of microaneurysm and early diagnosis system for non-proliferative diabetic retinopathy based on deep learning algorithms. IEEE Access.

[73] Hacisoftaoglu R.E., Karakaya M., Sallam A.B. (2020). Deep learning frameworks for diabetic retinopathy detection with smartphone-based retinal imaging systems. Pattern Recognit. Lett..

[74] Nawaz F., Ramzan M., Mehmood K., Khan H.U., Khan S.H., Bhutta M.R. (2021). Early Detection of Diabetic Retinopathy Using Machine Intelligence through Deep Transfer and Representational Learning. CMC Comput. Mater. Contin.

[75] Mishra S., Hanchate S., Saquib Z. Diabetic Retinopathy Detection using Deep Learning. Proceedings of the 2020 International Conference on Smart Technologies in Computing, Electrical and Electronics (ICSTCEE).

[76] Das S., Kharbanda K., Suchetha M., Raman R., Dhas E. (2021). Deep learning architecture based on segmented fundus image features for classification of diabetic retinopathy. Biomed. Signal Process. Control.

[77] Li X., Hu X., Yu L., Zhu L., Fu C.W., Heng P.A. (2020). CANet: Cross-disease Attention Network for Joint Diabetic Retinopathy and Diabetic Macular Edema Grading. IEEE Trans. Med. Imaging.

[78] Bodapati J.D., Shaik N.S., Naralasetti V. (2021). Composite deep neural network with gated-attention mechanism for diabetic retinopathy severity classification. J. Ambient. Intell. Hum. Comput..

[79] Hsieh Y.T., Chuang L.M., Jiang Y.D., Chang T.J., Yang C.M., Yang C.H., Chan L.W., Kao T.Y., Chen T.C., Lin H.C. (2021). Application of deep learning image assessment software VeriSee™ for diabetic retinopathy screening. J. Formos. Med Assoc..

[80] Wang J., Luo J., Liu B., Feng R., Lu L., Zou H. (2020). Automated diabetic retinopathy grading and lesion detection based on the modified R-FCN object-detection algorithm. IET Comput. Vis..

[81] PAhmad P., Jin H., Alroobaea R., Qamar S., Zheng R., Alnajjar F., Aboudi F. (2021). MH UNet: A Multi-Scale Hierarchical Based Architecture for Medical Image Segmentation. IEEE Access.

[82] Yang J.-J., Li J., Shen R., Zeng Y., He J., Bi J., Li Y., Zhang Q., Peng L., Wang Q. (2016). Exploiting ensemble learning for automatic cataract detection and grading. Comput. Methods Programs Biomed..

[83] (2018). Cataracts. https://www.mayoclinic.org/diseases-conditions/cataracts/diagnosis-treatment/drc-20353795.

[84] Lin Y., Zhang H., Hu G. (2018). Automatic retinal vessel segmentation via deeply supervised and smoothly regularized network. IEEE Access.

[85] Kumar D., Taylor G.W., Wong A. (2019). Discovery Radiomics With CLEAR-DR: Interpretable Computer Aided Diagnosis of Diabetic Retinopathy. IEEE Access.

[86] Kathiresan S., Sait A.R.W., Gupta D., Lakshmanaprabu S.K., Khanna A., Pandey H.M. (2020). Automated detection and classification of fundus diabetic retinopathy images using synergic deep learning model. Pattern Recognit. Lett..

[87] Goodfellow I., Bengio Y., Courville A. (2016). Deep Learning.

[88] Pal P., Kundu S., Dhara A.K. (2020). Detection of red lesions in retinal fundus images using YOLO V3. Curr. Indian Eye Res. J. Ophthalmic Res. Group.

[89] Gonzalez R.C. (2018). Deep convolutional neural networks [Lecture Notes]. IEEE Signal Process. Mag..

[90] Islam M.M., Yang H.C., Poly T.N., Jian W.S., Li Y.C.J. (2020). Deep learning algorithms for detection of diabetic retinopathy in retinal fundus photographs: A systematic review and meta-analysis. Comput. Methods Programs Biomed..

[91] Wang Z., Yang J. (2017). Diabetic retinopathy detection via deep convolutional networks for discriminative localization and visual explanation. arXiv.

[92] Malik H., Farooq M.S., Khelifi A., Abid A., Qureshi J.N., Hussain M. (2020). A Comparison of Transfer Learning Performance Versus Health Experts in Disease Diagnosis From Medical Imaging. IEEE Access.

[93] Ahn S., Pham Q., Shin J., Song S.J. (2021). Future Image Synthesis for Diabetic Retinopathy Based on the Lesion Occurrence Probability. Electronics.

[94] O’Shea K., Nash R. (2015). An introduction to convolutional neural networks. arXiv.

[95] Farooq S., Khan Z. (2019). A Survey of Computer Aided Diagnosis (Cad) of Liver in Medical Diagnosis. VAWKUM Trans. Comput. Sci..

[96] Sopharak A., Uyyanonvara B., Barman S. (2013). Simple hybrid method for fine microaneurysm detection from non-dilated diabetic retinopathy retinal images. Comput. Med Imaging Graph..

